# Plasma Metabolite Signature Classifies Male LRRK2 Parkinson’s Disease Patients

**DOI:** 10.3390/metabo12020149

**Published:** 2022-02-05

**Authors:** Chen Dong, Chandrashekhar Honrao, Leonardo O. Rodrigues, Josephine Wolf, Keri B. Sheehan, Matthew Surface, Roy N. Alcalay, Elizabeth M. O’Day

**Affiliations:** 1 Olaris, Inc., Framingham, MA 01702, USA; cdong@olarisbor.com (C.D.); chonrao@olarisbor.com (C.H.); lrodrigues@olarisbor.com (L.O.R.); jwolf@olarisbor.com (J.W.); ksanborn@gmail.com (K.B.S.); 2 Department of Neurology, Columbia University Irving Medical Center, New York, NY 10032, USA; surfacematt12@gmail.com (M.S.); rna2104@cumc.columbia.edu (R.N.A.); 3 Neurological Institute, Tel Aviv Sourasky Medical Center, Tel Aviv 6423906, Israel

**Keywords:** Parkinson’s disease, biomarker, metabolite, leucine, machine learning

## Abstract

Parkinson’s disease (PD) is a progressive neurodegenerative disease, causing loss of motor and nonmotor function. Diagnosis is based on clinical symptoms that do not develop until late in the disease progression, at which point the majority of the patients’ dopaminergic neurons are already destroyed. While many PD cases are idiopathic, hereditable genetic risks have been identified, including mutations in the gene for LRRK2, a multidomain kinase with roles in autophagy, mitochondrial function, transcription, molecular structural integrity, the endo-lysosomal system, and the immune response. A definitive PD diagnosis can only be made post-mortem, and no noninvasive or blood-based disease biomarkers are currently available. Alterations in metabolites have been identified in PD patients, suggesting that metabolomics may hold promise for PD diagnostic tools. In this study, we sought to identify metabolic markers of PD in plasma. Using a ^1^H-^13^C heteronuclear single quantum coherence spectroscopy (HSQC) NMR spectroscopy metabolomics platform coupled with machine learning (ML), we measured plasma metabolites from approximately age/sex-matched PD patients with G2019S LRRK2 mutations and non-PD controls. Based on the differential level of known and unknown metabolites, we were able to build a ML model and develop a Biomarker of Response (BoR) score, which classified male LRRK2 PD patients with 79.7% accuracy, 81.3% sensitivity, and 78.6% specificity. The high accuracy of the BoR score suggests that the metabolomics/ML workflow described here could be further utilized in the development of a confirmatory diagnostic for PD in larger patient cohorts. A diagnostic assay for PD will aid clinicians and their patients to quickly move toward a definitive diagnosis, and ultimately empower future clinical trials and treatment options.

## 1. Introduction

Parkinson’s disease (PD) is a chronic, progressive neurodegenerative disease that causes devastating loss of motor and nonmotor function over time. PD is the second-most common neurodegenerative disorder after Alzheimer’s dementia, with over 6 million people affected worldwide [1,2]. Currently, the diagnosis is based on clinical assessment of symptoms, and there are no blood-based biomarkers to diagnose the disease.

Postural instability, bradykinesia, rigidity, and resting tremor are the primary clinical indications suggestive of PD. Most patients do not exhibit these symptoms until 5–10 years after the neuropathology develops, at which point it is estimated that 80% of their dopaminergic neurons are lost [3]. Once symptoms are present, PD remains challenging to diagnose due to comorbidities such as cerebrovascular disease, dementia, and other neurological disorders [4]. Although resting tremor is a hallmark clinical feature of PD, not all patients with pathologically confirmed PD present with it. In clinically uncertain cases, DaTScan imaging, an FDA-approved imaging test, may be used to evaluate dopamine activity in commonly affected areas such as the striatum [5]. A pathologically confirmed PD diagnosis can only be obtained post-mortem by evaluating additional hallmarks such as loss of midbrain dopaminergic (mDA) neurons in the substantia nigra pars compacta (SNpc) and the presence of Lewy bodies containing aggregated alpha-synuclein protein filaments and/or hyperphosphorylated Tau protein [6]. However, post-mortem analyses have revealed that as many as 32% of patients with a pathologically confirmed PD diagnosis were misdiagnosed [7]. At present, therapies for PD, such as dopamine replacement therapy, aim to alleviate motor symptoms, and do not slow or reverse disease progression. Furthermore, patients treated with dopamine replacement therapy such as L-DOPA often develop L-DOPA-induced dyskinesia, an adverse treatment reaction occurring during advanced stages of PD [3,8].

Biomarkers of disease are urgently needed to reduce time to diagnosis and improve diagnostic accuracy, allowing patients to seek treatment while motor skills remain preserved. Further PD biomarkers are needed to enroll the appropriate patient populations in clinical trials and to more accurately assess disease-modifying therapies. As PD is widely recognized as a clinically and pathologically diverse group of disorders rather than a single disease, these markers also have the potential to improve diagnosis of subdisorders, development of precision therapeutics, and identification of underlying diverse disease mechanisms [9,10]. At present, several proposed PD biomarkers have failed to correlate with diagnosis and/or progression, including perturbed dopamine metabolites, decreased levels of alpha-synuclein in the cerebrospinal fluid and elevated levels of oligomeric alpha-synuclein in the plasma [11], gait-cycle irregularities [12], and isotope tracing to the SNpc [13]. Thus, there remains a critical unmet need to identify biomarkers to detect PD at its earliest onset.

PD is a multifactorial disorder; males are 1.4 times more likely to develop PD, and age, environment, and genetic factors contribute to disease development [8]. Although 90% of PD cases are idiopathic, ~10% of cases are related to known genetic risk factors [14], and overall hereditability is estimated to be ~20% for some populations [15]. Several genetic risk factors have been identified, including mutations in alpha-synuclein (SNCA), Parkin (PRKN), DJ-1 (PARK7), PTEN-induced kinase (PINK1), and leucine-rich repeat kinase 2 (LRRK2) protein, as well as glucocerebrosidase (GBA) and other lysosomal enzymes [6]. Patients with genetically linked PD who undergo regular screening are an important population to uncover biomarkers associated with disease progression, as the patients are often aware they are at risk for the disease before developing symptoms. 

PD-causing LRRK2 mutations are the greatest known cause of heritable PD, representing approximately 10% of autosomal familial dominant PD, and have also been implicated in 1–3% of sporadic PD cases. The prevalence of familial and sporadic Parkinsonism due to LRRK2 mutations ranges from 0.1–41%, varying by ethnic population, with the highest prevalence of cases found in patients from North Africa (30–40%) and Ashkenazi Jews (10–20%) [16,17,18]. Presentation of clinical symptoms in LRRK2 PD carriers is described as similar, if not indistinguishable, from sporadic PD patients [19,20].

LRRK2 is a large multi-domain enzyme expressed in diverse tissues and cell types that plays a role in many signal transduction pathways within the cell [16,21]. LRRK2 is a member of the ROCO protein family, which is characterized by a GTPase Ras-like G domain (Roc) followed by a C-terminal of Roc (COR) domain. LRRK2 also contains a serine-threonine kinase domain and can regulate several proteins by phosphorylation [22]. LRRK2 signaling has been shown to influence autophagy, mitochondrial function, transcription, molecular structural integrity, the endo-lysosomal system, and the immune response [16,21]. Although its complete function remains unknown, LRRK2 is predicted to play an important role in WNT/beta-catenin, Rab, and WNT/PCP signaling pathways [14]. In addition to PD, LRRK2 mutations also play a role in systemic lupus erythematosus, Crohn’s disease, inflammatory bowel disease, leprosy, and cancer [16]. Thus, better understanding of LRRK2’s function has the potential to affect efforts to target a wide range of human diseases.

Glu2019Ser (G2019S), the most prevalent LRRK2 mutation, has been shown to enhance LRRK2 kinase activity, perturbing mitochondrial bioenergetics and dynamics and leading to increased reactive oxygen species (ROS) within the cell [1,8,16]. A recent study demonstrated that PD patients without a LRRK2 mutation also show increased LRRK2 activity, suggesting that LRRK2 may be a common downstream node affected in PD [23]. However, not all persons with a LRRK2 mutation develop PD, and efforts are underway to understand the mutation penetrance [24]. Global metabolic profiling has the potential to highlight the imbalance of metabolic homeostasis driven by LRRK2 mutation and shed light on the role of this key protein in PD development.

Metabolomics is the comprehensive analysis of low-molecular-weight molecules that provide energy and biomass for life to exist [25]. Alterations in metabolism have been linked to several diseases, including cancer, diabetes, metabolic disorders, and neurodegeneration [3]. Understanding differences between the metabolic profiles of individuals or groups of healthy or diseased persons can shed light on the pathways underlying a disease process. Differences between healthy individuals and those who develop a disease are a result of a combination of factors, including genetic background, epigenetic modifications, lifestyle, stress, and environment [26,27]. Metabolic profiling elucidates the complex interaction between genes and the internal and external environment. Evaluating the metabolic profile of samples from PD patients and non-PD controls has the potential to identify candidate biomarkers of PD, creating a “fingerprint” for early diagnosis and prognosis, allowing clinicians to monitor disease progression and the effect of disease-modifying therapy.

Several small studies have identified metabolite changes in different cohorts of patients with PD, including changes to branched amino acids, fatty acids, and antioxidant levels [28]. One study found that bis(monoacylglycerol)phosphate isoforms were increased in the urine of patients with LRRK2 mutation independent of PD status, and that among PD patients, total levels correlated with cognitive decline [29]. In a separate study comparing PD patients with G2019S-LRRK2 mutations, asymptomatic family members with or without G2019S mutations, patients with idiopathic PD, and non-PD controls, patients with PD had significantly lower levels of urate in their blood than non-PD controls or asymptomatic LRRK2 mutation carriers [11], a result later validated by a larger study [24]. Urate is an endogenous purine metabolite with antioxidant and neuroprotectant properties [24]. One hypothesis suggests that elevated urate in non-PD patients serves as a neuroprotectant to reduce the risk and rate of disease [30]. Unfortunately, a randomized clinical trial evaluating urate-elevating inosine treatment in PD was terminated early due to lack of effect, which suggests that other factors may play a role in PD progression [31]. New strategies are needed to comprehensively analyze metabolites differentially expressed in PD and link them back to the underlying biology. 

We have previously demonstrated that using a global, unbiased, multi-dimensional ^1^H-^13^C heteronuclear single quantum coherence spectroscopy (HSQC) NMR spectroscopy metabolomics platform coupled with machine learning (ML) techniques, we are able to uncover metabolite-based biomarkers that correlate with disease status and response to therapy [32,33,34]. Further, as metabolomics studies are widely acknowledged to suffer from reproducibility challenges [35,36,37,38], we documented the reproducibility of our platform and how to translate our results for vitro diagnostic development [25,32,33]. In this study, we used our NMR and ML platform to measure plasma metabolites from approximately age/sex-matched PD patients with G2019S LRRK2 mutations and non-PD controls wild-type for LRRK2. The final model predicted PD with 79.7% accuracy, 81.3% sensitivity, and 78.6% specificity in male patients with LRRK2 mutations. The differential metabolites identified had links to known biological changes occurring during PD development. Our metabolomics-based biomarker platform has the potential to improve the diagnosis for PD, which is a critical first step towards treating and ultimately curing the disease. 

## 2. Results

### 2.1. LRRK2 PD Patient Sample Characteristics

Metabolites were extracted from plasma samples collected from patients diagnosed with PD with a LRRK2 G2019S mutation (N = 46 cases) and non-PD approximately age/sex-matched controls without LRRK2 mutations (N = 65 controls) (Table S1). All samples were assessed for quality assurance and control (QA/QC) by measuring the mean, median, maximum, and minimum intensity values and the number of metabolite resonances detected (Figure S1). One outlier sample (patient ID 15) was excluded, as it had severe streaking and significantly lower mean intensity value than other samples, which can be a sign of sample degradation (Figure S2). The filtered sample set was enriched for male samples (71% LRRK2 PD and 64% control). As sex is known to influence metabolite levels [39], we conducted analysis for both sexes together, as well as males and females separately (Table S2). Based on the small number of differential features for both the unstratified and the female analysis, as well as the reduced size of the female cohort (N = 13 cases and N = 22 controls), all additional analysis focused on male subjects, with N = 32 male LRRK2 PD patients and N = 42 male non-PD controls (Table 1). In the LRRK2 PD cohort, only 2 out of the 32 samples were collected from patients with a post-mortem confirmatory diagnosis (patient ID 47 and 57). The mean age at sample collection for the male LRRK2 PD patients was 70.0 ± 8.1 years, and the non-PD controls were slightly younger, but not statistically significant, with an average age of 69.5 ± 7.2 years (*p*-value = 0.780). In the male LRRK2 PD cohort, age at PD onset had a broad range, from 37 years old to 74 years old, with an average of 58.9 ± 10.1 years. In addition, the date at which the samples were collected after PD diagnosis ranged from 0.0 to 28.2 years, with an average of 10.9 years. The Montreal Cognitive Assessment (MoCA) score and body mass index (BMI) for the LRRK2 cohort was lower than, but not statistically significant for, the controls, 25.4 vs. 26.5 (*p*-value = 0.206) and 26.7 vs. 28.3 (*p*-value = 0.090), respectively. All cases and most controls (92.9%) were white, but not statistically different in ethnicity (*p*-value = 0.304). Additional metadata on treatment interventions such as brain surgery were provided (Table S3). In addition to patient clinical data, sample storage conditions, such as length of time in a freezer, can impact metabolomic results [40]. The majority of the samples (85.1%) had freezer time under 5 years (Table S4), and those with longer freezer time were closely monitored.

### 2.2. Differential Metabolites in Male LRRK2 PD

**Figure 1:**
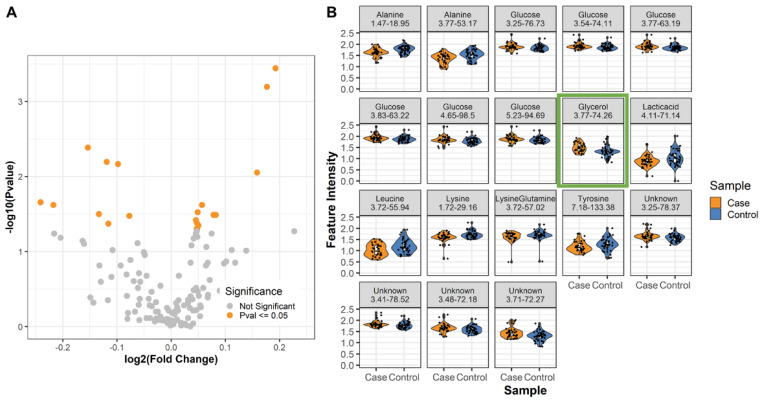
Differential metabolites correlated with male LRRK2 PD. (**A**) Volcano plot of the metabolite resonances extracted from the plasma of male LRRK2 PD patients and non-PD controls, with orange dots indicating significantly different resonances (*p* < 0.05). (**B**) Violin plots of the significant resonances identified in (**A**). The resonance at 3.77 and 74.26 ppm in the ^1^H and ^13^C dimensions indicated by the green box passed the FDR *p*-value of <0.05.

Using a Kruskal–Wallis (KW) test for significance, we identified 18 metabolite resonances with intensity values that were significantly different (*p* < 0.05) between male cases and controls (Figure 1A,B), in which 11 resonances were increased and 7 decreased in the PD patients compared to the non-PD controls. While not a direct 1:1 relationship due to transfer of magnetization and other factors, the intensity for each resonance can be used as a proxy for relative concentration. Likely due to the limited sample size, only 1 resonance passed the false discovery rate (FDR) correction (FDR *p*-value < 0.05, Figure 1B, green box). 

The differential metabolite resonances were matched by chemical shift to reference libraries [40] of known metabolites for annotation (Table 2, and Figure S3). Each resonance represents a carbon atom attached to a unique proton (a C-H pair), and thus it is possible that the differential resonances map to the same metabolite. For example, glucose and alanine had 6 and 2 matching resonances, respectively. Other differential resonances were mapped to lysine/glutamine, leucine, tyrosine, lactate, and glycerol. Resonances from differential metabolites that would require experimental validation to distinguish matching-ambiguities (“unknown” features) were kept for further analysis and referenced by their chemical shift position. The average fold change between PD patients and controls was 1.2. 

### 2.3. Metabolite Pathway Analysis

**Figure 2:**
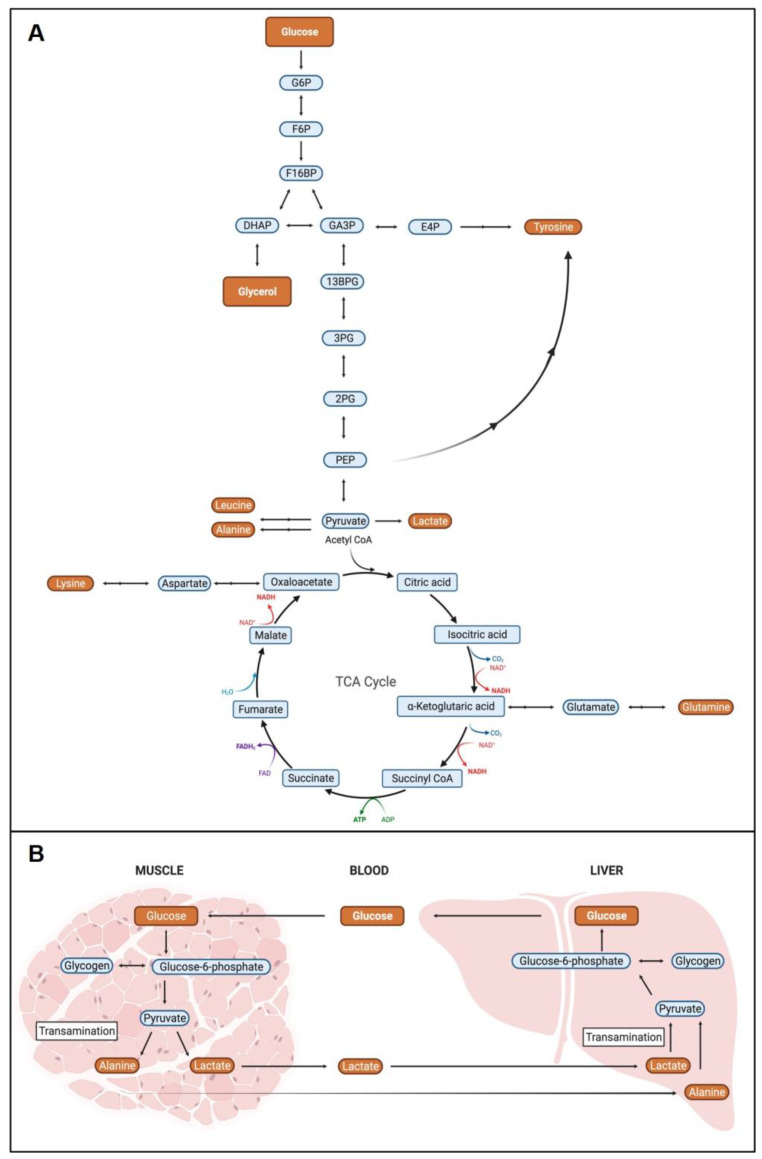
Dysregulated Male LRRK2 PD Metabolic Pathways. (**A**) Altered metabolites in male LRRK2 PD are enriched for amino acids and metabolites associated with glycolysis and gluconeogenesis. (**B**) Altered metabolites glucose, alanine, and lactate are central components of the glucose-alanine and Cori cycle important for supporting gluconeogenesis in the liver. Detected differential metabolites are highlighted in orange boxes. Double arrows represent multiple enzymatic transformational steps. Abbreviations: G6P; Glucose-6-phosphate, F6P; Fructose-6-phosphate, F16BP; Fructose 1,6-biphosphate, DHAP; dihydroxyacetone phosphate, GA3P; Glyceraldehyde-3-phosphate, 13BP; 1,3-Biphosphoglycerate, 3PG; 3-Phosphoglycerate, 2PG; 2-Phosphoglycerate, PEP; Phosphoenolpyruvate.

The dysregulated metabolites in the PD patients were enriched for amino acids and metabolites associated with glycolysis and gluconeogenesis. Many of the metabolites can be interconverted through known metabolic pathways (Figure 2A). For example, pyruvate, the end product of glycolysis, can be directly converted to lactate and used to generate alanine or supply the citric acid cycle (TCA) to produce additional amino acids such as glutamine. Glycolysis also generates 3-phosphoglycerate, which is a building block for the production of erythrose 4-phosphate, a precursor for tyrosine biosynthesis. Similarly, glycerol, identified as increased in PD patients, can be used to generate glucose and/or other glycolytic intermediates by conversion into dihydroxyacetone phosphate. Leucine and lysine are essential amino acids and must be obtained through diet [41].

Many of these metabolites have been previously identified as altered in PD [42], suggesting that flux through these pathways could be an important contributor to PD biology. This analysis was performed on plasma; thus, it is also important to consider how circulating metabolite levels affect the PD biology. We identified glucose, alanine, and lactate as altered metabolites in PD patients. These metabolites form part of the glucose–alanine and Cori cycle, wherein in organs like skeletal muscle increase glycolysis and protein catabolism to drive the production of lactate and alanine, both of which easily diffuse to the liver to become substrates for gluconeogenesis (Figure 2B). This helps increase glucose availability in the blood, especially during times of stress [43]. Investigating the impact of these processes in LRRK2 PD and PD warrants further research in additional patient cohorts and will be a focus of future efforts. In addition, we are working with collaborators to test the significance of metabolic flux through these pathways in animal models of the disease, as they could point to new therapeutic targets for intervention.

### 2.4. Building a Male LRRK2 PD Classifier 

**Figure 3:**
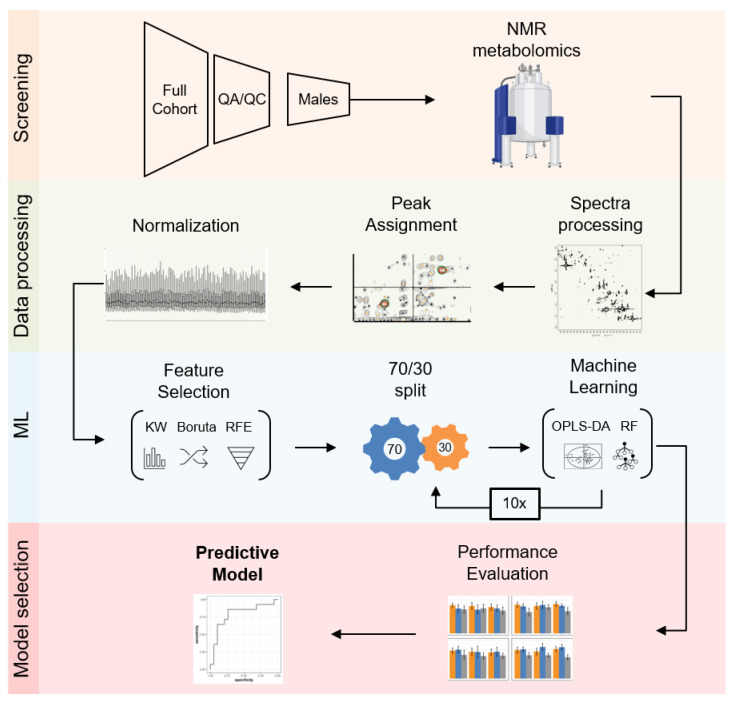
Classifier workflow. Following sample preparation and NMR filtering, raw spectra were processed, resonance peaks assigned to the database-matching metabolites, and the resonances across samples normalized. Modeling comprised feature selection and 10× cross-validation of OPLS-DA and RF methods. The model with the highest stability and overall performance was selected as the champion model.

The process that generated an accurate model to classify case from control patients comprised generating pre-defined feature sets, evaluating each set in distinct cross-validated machine learning (ML) algorithms, and selecting the final model based on performance in the training and test datasets (Figure 3). The pre-defined feature sets included: “Known”, differential metabolites resonances assigned to a unique metabolite; “Unknowns”, differential metabolite resonances that require experimental clarification; and “Clinical”, available sample-donor clinical information (Table S5). In addition to the KW test, feature sets were further subjected to 2 selection methods, Boruta, that selects features based on the Random Forest (RF) importance of the unchanged vs. shuffled feature [44], and RFE (Recursive Feature Elimination), that recursively eliminates the least important feature of each retrained model. Combinations of the feature sets (“Known”, “Unknown” and “Clinical”) with the feature selection methods (KW-only, KW-Boruta and KW-RFE) led to 12 feature sets for ML analysis (Table S6). The data were then split 70/30 into training and test data, 10× cross-validated, and assessed using orthogonal partial least squares discriminate (OPLS-DA) and RF models (Figure 3).

Cross-validation models were assessed based on accuracy, sensitivity, specificity, precision, and area under the curve (AUC) to distinguish LRRK2 PD patient samples from those of non-PD controls. Model selection started with comparing the performance and stability of the cross-validated ML models between the training and test splits (Figure S4 and Table S7). A pre-evaluation of the models excluded 10 unstable, and potentially overfitting, models because at least one of the five performance metrics was significantly different between the training and test datasets (*p* < 0.05, Student’s *t*-test).

For the remaining 14 models, the average cross-validated accuracy, sensitivity, specificity, precision, and AUC for all models were 67.6 ± 5.1%, 59.8 ± 7.1%, 73.3 ± 5.5%, 63.8 ± 5.7%, and 70.9 ± 2.2%, respectively. Comparing models using “Known” vs. “Known + Unknown” features, we observed improved testing AUC from 69.6 ± 0.5% to 71.8 ± 2.4% with the addition of “Unknown” features. Similarly, upon adding clinical data to the “Known” feature set, the testing AUC improved to 73.1 ± 3.1%. However, when adding clinical data to the “Known + Unknown” feature sets, the testing AUC dropped to 70.3 ± 3.0%, similar to the model performance using “Known” features. On average, OPLS-DA models had better performance than RF models, with 71.7 ± 2.1% and 70.3 ± 2.2% in AUC, respectively. Boruta and RFE selected features showed improved classification in RF models, but not in OPLS-DA models. In RF analysis, KW-Boruta and KW-RFE increased the AUC from 67.5 ± 14.3% (KW-only) to 69.0 ± 0.0% (KW-Boruta) and 71.9 ± 1.8% (KW-RFE). However, in OPLS-DA models, AUC decreased from 75.0 ± 8.6% (KW-only) to 70.4 ± 0.8% (KW-Boruta) and 73.3 ± 6.1% (KW-RFE).

**Figure 4:**
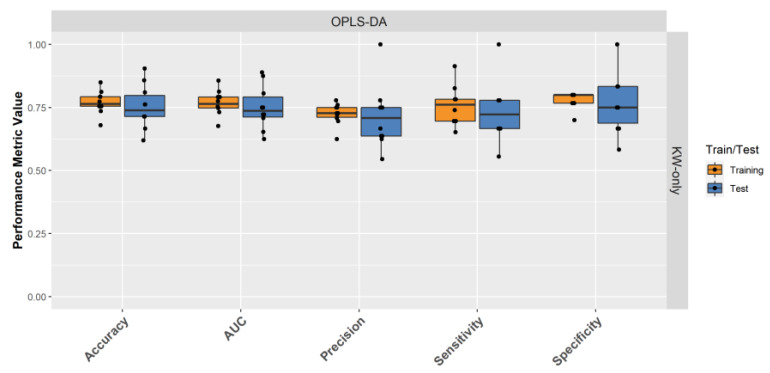
Cross-validation performance of champion machine learning algorithm for LRRK2 PD classification. The data with KW-only and “Known + Unknown” features was split 70/30 into training and test data, 10× cross-validated using an OPLS-DA model.

Following model ranking based on cross-validated stability and performance, and model-section based on the cross-validated AUCs, an OPLS-DA model using KW-only and “Known + Unknown” features was selected as the champion model. The OPLS-DA cross-validation model had 77.0 ± 4.6% accuracy, 75.7 ± 7.7% sensitivity, 78.0 ± 3.2% specificity, 72.4 ± 4.3% precision, and 76.8 ± 4.9% AUC for the training data, and 75.2 ± 8.6% accuracy, 73.3 ± 11.9% sensitivity, 76.7 ± 11.7% specificity, 71.4 ± 12.5% precision, and 75.0 ± 8.6% AUC for the test data (Figure 4). 

In addition, sensitivity analysis evaluated the effect of each metabolite on the champion model performance (Table S8). Removing any metabolite led to decreased AUC. Removing two resonances from alanine caused the most significant drop in AUC from 85% to 60.8%. This was followed by loss of two lysine resonances and then loss of all six glucose peaks, which dropped AUC to 64.6% and 65.4%, respectively. Withholding any of the single resonance identified from glycerol, tyrosine, lactic acid, and leucine dropped AUC to ~70%. Taken together, the sensitivity analysis demonstrated that all resonances are important for the accuracy of the model. Furthermore, it demonstrates that metabolites like glucose, which contributed six resonances, were not overly biasing the model, as other metabolites (alanine and lysine) more strongly influenced the ML metrics.

**Figure 5:**
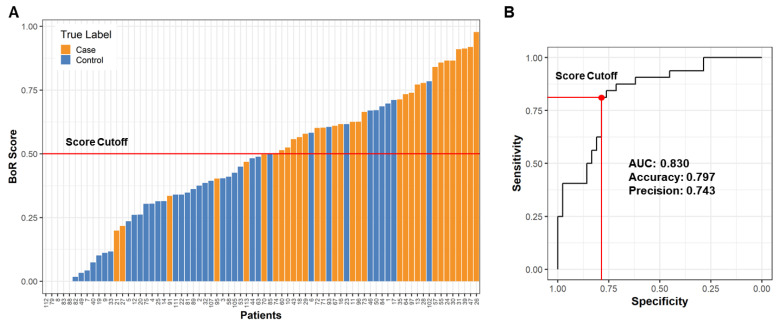
Olaris BoR score has high accuracy to classify male LRRK2 PD. (**A**) Waterfall plot of BoR score for all data with predicted cases scoring over 0.5 and predicted controls scoring under 0.5. True cases are colored orange and true controls are colored blue. (**B**) Receiver operator characteristic (ROC) analysis with 0.830 area under the curve (AUC). Red lines mark corresponding 81.3% sensitivity and 78.6% specificity using a score cutoff at 0.5.

Following selection of the champion model, OPLS-DA rankings were used to derive a proprietary Olaris^®^ BoR (biomarker of response) score that ranges from 0 to 1, with PD samples scoring over 0.5 and non-PD controls under 0.5 (Figure 5A). Once the BoR score was applied to the full dataset, the LRRK2 PD classifier had 79.7% accuracy, 81.3% sensitivity, 78.6% specificity, 74.3% precision and 83.0% AUC (Figure 5B). 

From the non-PD control population, one patient (ID 102) had a high BoR score suggestive of PD (Figure 5A). This patient had a relatively low MoCA score of 22 compared to the average of controls of 26.5, suggesting the patient could have mild cognitive impairment (Table S3). Similarly, there were two PD patients that had low scores suggestive of a non-PD status (ID 21 and 27). There were not any available clinical data supporting the low score, and neither of these two patients had a post-mortem confirmatory PD diagnosis. Even though there were only two patients with post-mortem confirmatory diagnosis, we were very encouraged to see they were predicted correctly and ranked 2nd (BoR 0.92) and 8th (0.84) highest in BoR scores. In addition we applied this model to the female patients, but as expected, it had poor overall accuracy (51.4% Figure S5), suggesting the importance to examine the influence of sex on metabolites. 

## 3. Discussion

PD is a growing concern, with a prevalence increasing more rapidly than other neurological disorders [8]. There is an unmet clinical need for improved diagnosis of PD to enable either early detection or greater confidence in diagnosis. While there are currently limited treatments available for PD, efforts are underway to develop new therapies. For these treatments to be successful, improved PD diagnostics is a prerequisite.

PD is influenced by aging, genetic predisposition, and exposure to exogenous and endogenous toxins [45]. While it was previously considered to be primarily a central nervous system disorder, PD is now understood to be multifactorial, with multiple mechanisms contributing to its pathogenesis. As metabolomics has been used to closely examine PD, PD may even be considered a metabolic disorder, with metabolic dysregulation contributing to neurodegeneration [1]. Previous studies have identified metabolites altered in older adults with PD, including elevation of circulating β-amino butyric acid, cystine, ornithine, phosphoethanolamine, and proline [46], and altered bacterial composition in patients with PD may be linked with changes in bacterial metabolites that affect patient health [47]. 

In this study, we utilized multi-dimensional NMR spectroscopy and ML to develop a classifier for LRRK2 PD. Focusing on male patients with G2019S LRRK2 mutations, we identified 18 significantly differential metabolite resonances between male LRRK2 PD patients and controls. Although the metabolites identified in our analysis have been previously linked to PD, many of the studies were performed in different biofluids, in both sexes and using different analytical techniques, making it difficult to directly compare results between studies. Nonetheless, there were many consistencies, such as the altered glucose metabolism in patients with PD [48,49,50]. We also observed an increase in glycerol levels in PD patients with LRRK2 mutations, consistent with previous reports, suggesting that glycerol phospholipid metabolism is upregulated in the plasma of PD patients [51]. Finally, we observed a decrease in lactate, which was similarly observed in the CSF of patients with PD [52]. Many of the altered metabolites, including lactate, alanine, and glutamine, are connected via known metabolic pathways, and differences in their levels may reflect dysregulation of these pathways overall in PD. 

We found that several amino acids, including alanine and lysine, were decreased in LRRK2 PD plasma samples. Similarly, in previous studies, alanine was found to be decreased in both serum and CSF of PD patients [52,53], and lysine levels were decreased in the CSF of PD patients [54]. Our data thus provide independent confirmation of these markers in a novel patient cohort, which is encouraging given the reproducibility concerns facing metabolomics studies. In contrast, our analysis showed that leucine and tyrosine were also decreased in LRRK2 PD plasma samples, while previous studies suggested that leucine is increased in the CSF, fecal and urine samples of patients with PD [55,56,57] and increased tyrosine levels were found in the CSF, urine, and saliva of PD patients [55,58,59]. Dysregulation of the synthesis of dopamine from tyrosine in PD has been well established [60], however it is possible that both tyrosine and leucine levels may be altered in different directions in different biofluids. Further, leucine and tyrosine have both been demonstrated to be elevated in people with metabolically unhealthy obesity [61], and while adding BMI did not improve our model accuracy, it is still possible that obesity and associated comorbidities may act as confounders in our analysis. Additionally, our study was restricted to LRRK2 PD, while previous studies examined levels of these metabolites in idiopathic PD, and it is unclear if genetic forms of PD are a reporter for all cases of PD. Finally, it is not unexpected to find some discordance due to reproducibility challenges in metabolomics, the observed small fold-change between PD patients and controls in our studies and others, and the sensitivity of metabolites to sex, diet, medication, and other factors. This underscores the need for additional controlled validation studies. Finally, while other studies observed decreased urate levels in LRRK2 PD plasma samples, we were unable to measure urate [11,24], as it lacks ^1^H-^13^C resonances. 

Glucose, alanine, and lactate were the primary metabolites altered in patients with PD. These metabolites form part of the glucose-alanine cycle and the Cori cycle (also known as the lactic acid cycle), which are central to gluconeogenesis, increasing glucose bioavailability and providing ATP to cells during times of stress or exertion. Interestingly, the glucose–alanine cycle (both hits in our analysis) has also been shown to be downregulated in PD patients [62]. This may be particularly relevant in our patient cohort given the established role of LRRK2 in oxidative stress and the effect of the G2019S mutation on ROS production and neuronal cell death in vitro [63,64]. Further research will focus on better understanding the role of these pathways in LRRK2-related PD and PD overall.

Using metabolomics data from this cohort of PD patients with LRRK2 mutations, we developed an OPLS-DA model to derive a biomarker-based score for PD. Although the current BoR score is based on limited data from a heterogeneous patient cohort and must be considered a first step towards a more comprehensive model, the score differentiated LRRK2 PD patients from controls with 79.7% accuracy, 81.3% sensitivity, 78.6% specificity, 74.3% precision and 83.0% AUC. While there were a few patients and controls who fell outside the score cutoff for non-PD vs. PD status, the majority were correctly predicted using the score. Furthermore, the two patients with a post-mortem biopsy-proven PD diagnosis were classified correctly. In this study, performance stability was used to rank the predictive models and a conservative BoR cutoff (0.5) was selected. Future studies on larger cohorts should also explore models and BoR cutoffs in which the balance of specificity and sensitivity is refined towards maximizing true positive or true negative predictions. 

Despite the encouraging first step towards a non-invasive confirmatory diagnostic, appropriate caution must be applied regarding the generalizability of these results to additional PD cohorts due to the limitations of this study. While we were able to build a classifier with high predictive accuracy to classify LRRK2 PD patients from non-PD controls, it remains to be determined if this signature classifies other familial or idiopathic forms of PD, especially as differences in omics results have been observed between familial vs. idiopathic PD [65]. Additionally, it is unclear whether the classifier can distinguish individuals with a LRRK2 mutation that do not manifest symptoms of PD. Moreover, although the analyzed cohort was approximately age- and gender-matched, we were not able to control for additional potential confounding factors such as medications or diet. Hence, it is possible that the PD LRRK2 BoR score may have bias towards some of these confounders. Full access to the patients’ clinical information would be necessary to address this limitation. 

Finally, this study utilized samples from a single relatively small cohort of LRRK2 PD patients, collected at various time points post diagnosis. The varied interval between the date of PD onset and sample collection is a concern. Some patients provided plasma samples upon diagnosis, while others had been suffering from disease for 28.2 years. This suggests that the “case” cohort had patients at various stages of disease progression, which could obscure differences, and might explain why the addition of clinical data such as MoCA and BMI did not add value to the model performance. Furthermore, analysis of samples collected prior to diagnosis would improve the ability of the BoR score to diagnose asymptomatic and early PD onset. While the model developed was built to account for the large amount of variability, further research will focus on lowering patient and sample variability by recruiting patients within 2–5 years of PD diagnosis; including samples collected prior to diagnosis in analysis; requiring, for example, fasting of patients prior to sample collection; and ensuring access to clinical data. The metabolite and ML pipeline described here provides a foundation to develop a confirmatory diagnostic for PD. 

## 4. Materials and Methods

The NMR reference standard, deuterated 3-(trimethylsilyl)-1-propanesulfonic acid sodium salt (DSS-d6, 98%) and deuterium oxide (D2O, 99.0%) were purchased from Cambridge Isotope Laboratory, Andover, MA, USA. Potassium dihydrogen phosphate (KH_2_ PO_4_) and dipotassium hydrogen phosphate (K_2_HPO_4_) were purchased from Millipore-Sigma (St. Louis, MO, USA).

### 4.1. Patient Enrollment and Plasma Collection

The study included 46 PD patients and 65 controls who participated in the ‘Spot’ study [66,67]. In brief, the Spot study included PD patients and genetically unrelated controls (mostly spouses) from the Center for Parkinson’s Disease at Columbia University Irving Medical Center in New York, NY, recruited between 2010–2021. All participants have been fully sequenced for GBA mutations and screened for the LRRK2 G2019S mutation. Information on demographics, medical history, medication, family history of PD, the Unified Parkinson’s Disease Rating Scale (UPDRS) in the “on” state and the Montreal Cognitive Assessment (MoCA) were collected from all participants. A 10cc EDTA tube was used to collect blood, which was centrifuged and aliquoted to 1cc plasma aliquots within 60 min of collection. Participants were non-fasting at the time of the blood donation. Samples were stored in a −80 °C freezer until processing. All study procedures were approved by the Columbia University IRB, and all participants signed informed consent.

### 4.2. NMR Sample Preparation 

Metabolites were extracted from 1 mL of human plasma via a methanol and chloroform liquid–liquid extraction. The aqueous phase was transferred to a Falcon tube and freeze-dried. Lipid extracts (0.25 mL) were isolated, dried, and stored at −80 °C for separate lipidomic analysis. The powder was reconstituted in 0.18 mL of 50 mM phosphate buffer at pH 7.4 in D_2_O and then immediately transferred to a 3 mm NMR tube for NMR data collection. The NMR standard, DSS-d6, was added to each sample for chemical shift referencing.

### 4.3. NMR Data Collection and Processing 

All NMR data were acquired on a Bruker AVANCE II solution-state NMR spectrometer equipped with a liquid nitrogen-cooled Prodigy TXI Cryoprobe operating at a proton frequency of 600 MHz using hsqcetgpsisp2.2 pulse program with other parameters: 90° pulse angle, 8.53 μs pulse width, 1.5 s relaxation delay, 128 dummy scans, 203 receiver gain and 36 number of scans. The 1JCH used was 145 Hz (1.72 ms). The number of complex points was set to 512 and 32 (25% NUS) for direct and indirect dimension, respectively. The spectral width of 15.97 ppm (9578.44 Hz) and 160.00 ppm (24131.775 Hz) were used for ^1^H and ^13^C, respectively. NUS schedules were generated using Poisson gap distribution [68], and spectral data were processed using the NMRPipe software package as previously described [69]. The NUS data was reconstructed using iterative soft thresholding according to the hmsIST algorithm [68]. Chemical shift queries, metabolite identifications and quantifications were performed using the COLMARm NMR webserver (http://spin.ccic.osu.edu/index.php/colmarm/index, accessed on 21 October 2021) [41]. Metabolite resonances with a signal intensity greater than 2 × 10^6^ (24× S/N) were dynamically binned into clusters using DBSCAN (Density-Based Spatial Clustering of Applications with Noise) in both ^1^H and ^13^C dimensions. Resonances below the signal intensity cut-off were imputed with a two-step process either by matching resonances between 1 × 10^6^ (12× S/N) and 2 × 10^6^ by setting those below the limit of detection to a constant value of 7 × 10^5^. Resonance clusters were normalized and filtered using a criterion of being present in at least 50% of all samples in one class.

### 4.4. Statistical Analysis

The distribution of metabolic resonance intensity was tested by Shapiro–Wilk normality test and Q-Q plot. Due to non-normal distribution, a Kruskal–Wallis (KW) non-parametric one-way analysis of variance (ANOVA) was used to test for significant differences in measured NMR resonances between groups of interest. The test of significance was also adjusted based on false discovery rate (FDR) for multiple hypothesis testing correction. Fold changes (FC) were calculated as the ratio of the mean intensities of the two groups. All statistical analyses and ML modeling were performed using R 4.1.1 (http://cran.rproject.org, accessed 19 November 2021) with the following packages: caret and Boruta for feature selection [44,70], randomForest and ropls for modeling [71,72], and ggplot2 and pROC for result visualization [73,74].

## 5. Conclusions

The process of diagnosis for Parkinson’s disease can be arduous, with an average time of 7 years from symptom onset until official diagnosis [75]. During this time, patients struggle through a frustrating and costly diagnostic odyssey, only to find limited treatment options. Early diagnostics stand to revolutionize PD care. While further research is needed to expand these results beyond a limited patient cohort, the Olaris^®^ BoR score for PD represents a substantial step forward in improving diagnostic precision for LRRK2 PD. The model relies on quantifying metabolomic signatures in the plasma of male LRRK2 PD patients, thus requiring only minimally invasive sample collection and providing rapid results. This novel scoring system may help clinicians and their patients to quickly move toward a definitive PD diagnosis, widening the critical window in which therapy is beneficial, allowing time for further research toward curative therapy, and providing PD patients with many more functional years.

## Figures and Tables

**Table 1:** Male LRRK2 patient sample characteristics.

		Case	Control
Number of samples		32	42
Age at sample collection in years (SD)		70.0 (8.1)	69.5 (7.2)
Age at onset in years (SD)		58.9 (10.1)	-
Sample collection time after PD Dx in years (SD)		10.9 (8.3)	-
MoCA (SD)		25.4 (4.8)	26.5 (2.5)
BMI (SD)		26.7 (2.6)	28.3 (4.8)
Ethnicity	White	32	39
	Hispanic	0	1
	Black	0	2

**Table 2:** Top significantly different ^1^H-^13^C HSQC metabolite resonances (*p*-value < 0.05) between male LRRK2 PD and controls.

Annotation	^1^H ppm	^13^C ppm	Fold Change (Case/Control)	*p*-Value	FDR *p*-Value
Glycerol	3.77	74.26	1.130	0.001	0.042
Alanine	3.77	53.17	0.899	0.004	0.178
Lysine	1.72	29.16	0.921	0.006	0.178
Alanine	1.47	18.95	0.934	0.007	0.178
Unknown	3.71	72.27	1.116	0.009	0.193
Lactic acid	4.11	71.14	0.846	0.022	0.302
Leucine	3.72	55.94	0.860	0.024	0.302
Unknown	3.41	78.52	1.040	0.024	0.302
Glucose	3.77	63.19	1.034	0.030	0.302
Tyrosine	7.18	133.38	0.912	0.032	0.302
Unknown	3.48	72.18	1.056	0.032	0.302
Unknown	3.25	78.37	1.059	0.032	0.302
Lysine/Glutamine	3.72	57.02	0.948	0.033	0.302
Glucose	3.54	74.11	1.032	0.038	0.302
Glucose	4.65	98.5	1.033	0.041	0.302
Glucose	3.25	76.73	1.035	0.045	0.302
Glucose	3.83	63.22	1.033	0.050	0.302
Glucose	5.23	94.69	1.034	0.050	0.302

## Data Availability

Data available on request subject to proprietary restrictions.

## Supplementary Materials

The following supporting information can be downloaded at: https://www.mdpi.com/article/10.3390/metabo12020149/s1, Table S1, Patient Characteristics; Table S2, Summary of Case vs. Control Analysis; Table S3, Male Patient Metadata; Table S4, Male Sample Freezer Time; Table S5, Pre-defined Feature Sets; Table S6, Feature Sets for ML Analysis; Table S7, Cross-validation ML Model Performance; Table S8, Sensitivity Analysis of Champion Model, Figure S1, NMR data quality control and assurance by measuring the mean, median, maximum and minimum intensity values and number of metabolite resonances detected; Figure S2, NMR spectral comparison; Figure S3, NMR annotations for top differential features; Figure S4, Performance of champion machine learning algorithm for LRRK2 PD classification; Figure S5, Receiver operator characteristic (ROC) analysis with 0.556 area under the curve (AUC) using male BoR score to predict on female cohort.
