# Supplementary material for: Plasma Metabolite Signature Classifies Male LRRK2 Parkinson’s Disease Patients
Source: Metabolites. 2022 Feb 5;12(2):149. doi: 10.3390/metabo12020149 (PMC8876175; doi:10.3390/metabo12020149)
Supplement: Supplementary file 1 [file metabolites-12-00149-s001.zip › metabolites-1546519-supplementary.pdf]

Table S1. Patient Characteristics

		Case	Control
N		46	65
Male (%)		33 (72%)	42 (65%)
Brain surgery (%)		8 (17%)	0 (0%)
Age at sample collection in years (SD)		71.4 (8.6)	68.5 (7.2)
Age at onset in years (SD)		60.3 (10.7)	NA
Sample collection time after PD Dx in years (SD)		10.9 (7.7)	NA
MoCA (SD)		25.4 (4.3)	26.7 (2.3)
BMI (SD)		25.6 (3.9)	27.4 (4.8)
Ethnicity	White	46	59
	Hispanic	0	2
	Black	0	2
	Asian or Pacific Islander	0	2

Table S2. Summary of Case vs Control Analysis

Analysis	Category	Features Tested	Features Passing P<0.05	Features Passing FDR-adjusted P<0.05
Unstratified	45Case 64Control	126	8	0
Female	13Case 22Control	126	6	0
Male	32Case 42Control	126	18	1

Table S3. Male Patient Metadata

Internal Patient ID	Disease Status	LRRK2 Status	Age at Sample Collection	Age at Onset	Brain Surgery	Collection Time after Dx years	Ethnicity	Hispanic?	BMI	MoCA
1	PD-	LRRK2-	71.6	NA	0	NA	White	No	23.0	27
2	PD-	LRRK2-	67.2	NA	0	NA	White	No	23.7	28
3	PD-	LRRK2-	67.1	NA	0	NA	White	No	32.1	27
4	PD-	LRRK2-	70.6	NA	0	NA	Black	No	33.9	24
5	PD-	LRRK2-	67.5	NA	0	NA	White	No	36.0	26
6	PD-	LRRK2-	67.8	NA	0	NA	White	No	24.4	29
7	PD-	LRRK2-	67	NA	0	NA	White	No	28.2	25
8	PD-	LRRK2-	68.8	NA	0	NA	White	No	29.3	25
9	PD-	LRRK2-	73	NA	0	NA	White	No	23.5	30
10	PD+	LRRK2+	79.6	58	0	21.6	White	No	26.5	28
11	PD+	LRRK2+	67	67	0	0	White	No	23.8	26
12	PD-	LRRK2-	70.5	NA	0	NA	White	No	39.2	26
13	PD+	LRRK2+	56.4	53	0	3.4	White	No	25.4	28
14	PD-	LRRK2-	63.3	NA	0	NA	White	No	23.6	25
16	PD+	LRRK2+	73.4	72	0	1.4	White	No	24.0	28
17	PD-	LRRK2-	67.9	NA	0	NA	White	No	20.1	27
18	PD+	LRRK2+	78.2	50	0	28.2	White	No	26.3	18
19	PD-	LRRK2-	70.9	NA	0	NA	White	No	29.7	28
20	PD-	LRRK2-	71.1	NA	0	NA	Hispanic	Puerto Rico	38.0	30
21	PD+	LRRK2+	70.6	65	0	5.6	White	No	26.6	22
22	PD-	LRRK2-	73	NA	0	NA	White	No	30.1	28
23	PD-	LRRK2-	75.3	NA	0	NA	White	No	27.9	24
24	PD+	LRRK2+	77.5	71	0	6.5	White	No	22.8	28
25	PD-	LRRK2-	67.6	NA	0	NA	White	No	30.6	25
26	PD+	LRRK2+	80.7	71	0	9.7	White	No	26.6	26
27	PD+	LRRK2+	70.7	62	0	8.7	White	No	32.1	24
28	PD+	LRRK2+	61.6	57	0	4.6	White	No	24.6	23
29	PD+	LRRK2+	59.3	56	0	3.3	White	No	25.7	28
30	PD+	LRRK2+	66.7	59	0	7.7	White	No	25.6	24
31	PD+	LRRK2+	73.8	53	0	20.8	White	No	25.1	30
32	PD-	LRRK2-	72.2	NA	0	NA	White	No	28.7	26
33	PD-	LRRK2-	72.7	NA	0	NA	White	No	26.8	26
35	PD+	LRRK2+	68.2	67	0	1.2	White	No	27.1	25
39	PD+	LRRK2+	81.4	74	0	7.4	White	No	29.0	8
40	PD-	LRRK2-	85.1	NA	0	NA	White	No	22.8	26
43	PD+	LRRK2+	63.4	37	0	26	White	No	27.5	28
44	PD-	LRRK2-	76.1	NA	0	NA	White	No	31.7	26
46	PD-	LRRK2-	73.4	NA	0	NA	White	No	36.9	28
47	PD+	LRRK2+	62.5	41	1	21	White	No	29.8	30
49	PD-	LRRK2-	65.6	NA	0	NA	White	No	23.4	29
50	PD-	LRRK2-	66.7	NA	0	NA	White	No	24.8	27
53	PD-	LRRK2-	79.6	NA	0	NA	White	No	23.1	27
55	PD+	LRRK2+	76.7	61	0	15	White	No	27.4	25
57	PD+	LRRK2+	66.9	51	0	15	White	No	29.2	26
58	PD-	LRRK2-	65.1	NA	0	NA	White	No	31.3	30
60	PD+	LRRK2+	63	44	1	18	White	No	24.8	29
63	PD-	LRRK2-	70	NA	0	NA	White	No	24.8	27
64	PD+	LRRK2+	76.5	66	0	10	White	No	32.8	27
67	PD+	LRRK2+	70.5	55	0	15	White	No	23.2	28
70	PD+	LRRK2+	77.6	57	1	20	White	No	23.2	19
71	PD+	LRRK2+	65.7	60	0	5	White	No	29.5	29
72	PD+	LRRK2+	58.4	37	1	21.4	White	No	27.9	29
73	PD+	LRRK2+	56.3	55	0	1	White	No	31.5	30
74	PD+	LRRK2+	87.8	66	1	21	White	No	26.3	18
75	PD-	LRRK2-	44.2	NA	0	NA	Black	No	35.9	29
79	PD-	LRRK2-	70.4	NA	0	NA	White	No	29.3	25
81	PD-	LRRK2-	73.2	NA	0	NA	White	No	21.9	28
82	PD-	LRRK2-	84.7	NA	0	NA	White	No	26.5	26
83	PD-	LRRK2-	74.4	NA	0	NA	White	No	25.8	23
84	PD-	LRRK2-	69.2	NA	0	NA	White	No	23.0	NA
85	PD-	LRRK2-	77.2	NA	0	NA	White	No	24.4	NA
88	PD-	LRRK2-	68.9	NA	0	NA	White	No	34.4	25
89	PD-	LRRK2-	67.9	NA	0	NA	White	No	30.5	19
91	PD+	LRRK2+	66.1	54	0	12.1	White	No	24.3	NA
93	PD-	LRRK2-	71.6	NA	0	NA	White	No	26.4	29
95	PD+	LRRK2+	66.8	66	0	0.8	White	No	25.8	NA
96	PD+	LRRK2+	63.7	56	1	7	White	No	25.0	27
97	PD+	LRRK2+	79	71	0	8	White	No	26.1	NA
102	PD-	LRRK2-	73.1	NA	0	NA	White	No	29.6	22
105	PD-	LRRK2-	50.2	NA	0	NA	White	No	25.1	27
107	PD-	LRRK2-	64.4	NA	0	NA	White	No	NA	21
111	PD-	LRRK2-	64.8	NA	0	NA	White	No	29.8	29
112	PD-	LRRK2-	58.2	NA	0	NA	White	No	28.6	30
113	PD+	LRRK2+	75.3	73	0	2	White	No	30.1	25

Table S4. Male Sample Freezer Time

	**Freezer time 0~5 years**	**Freezer time 5~7 years**	**Freezer time >7 years**
**Case**	28	2	2
**Control**	35	1	5

Table S5. Pre-defined Feature Sets

Known	Unknown	Clinical
Alanine_1.47_18.955<br>Alanine_3.772_53.166<br>Glucose_3.246_76.738<br>Glucose_3.538_74.093<br>Glucose_3.768_63.191<br>Glucose_3.831_63.217<br>Glucose_4.648_98.502<br>Glucose_5.234_94.693<br>Glycerol_3.772_74.329<br>Lactic_acid_4.109_71.139<br>Leucine_3.719_55.923<br>Lysine_1.72_29.156<br>Lysine_3.717_57.021<br>Tyrosine_7.181_133.38	Unknown_3.249_78.373<br>Unknown_3.406_78.52<br>Unknown_3.477_72.189<br>Unknown_3.712_72.274	Age_at_Sample_Collection<br>MoCA<br>BMI

Table S6. Feature Sets For ML Analysis

Known			Known_Unknown			Known_Clinical			Known_Unknown_Clinical		
**KW-only**	**KW_Boruta**	**KW-RFE**	**KW-only**	**KW_Boruta**	**KW-RFE**	**KW-only**	**KW_Boruta**	**KW-RFE**	**KW-only**	**KW_Boruta**	**KW-RFE**
Alanine_1.47_18.955<br>Alanine_3.772_53.166<br>Glucose_3.246_76.738<br>Glucose_3.538_74.093<br>Glucose_3.768_63.191<br>Glucose_3.831_63.217<br>Glucose_4.648_98.502<br>Glucose_5.234_94.693<br>Glycerol_3.772_74.329<br>Lactic_acid_4.109_71.139<br>Leucine_3.719_55.923<br>Lysine_1.72_29.156<br>Lysine_3.717_57.021<br>Tyrosine_7.181_133.38	Alanine_1.47_18.955<br>Alanine_3.772_53.166<br>Glycerol_3.772_74.329	Alanine_1.47_18.955<br>Alanine_3.772_53.166<br>Glucose_3.246_76.738<br>Glucose_3.538_74.093<br>Glucose_3.768_63.191<br>Glucose_3.831_63.217<br>Glucose_4.648_98.502<br>Glucose_5.234_94.693<br>Glycerol_3.772_74.329<br>Lactic_acid_4.109_71.139<br>Leucine_3.719_55.923<br>Lysine_1.72_29.156<br>Lysine_3.717_57.021<br>Tyrosine_7.181_133.38	Alanine_1.47_18.955<br>Alanine_3.772_53.166<br>Glucose_3.246_76.738<br>Glucose_3.538_74.093<br>Glucose_3.768_63.191<br>Glucose_3.831_63.217<br>Glucose_4.648_98.502<br>Glucose_5.234_94.693<br>Glycerol_3.772_74.329<br>Lactic_acid_4.109_71.139<br>Leucine_3.719_55.923<br>Lysine_1.72_29.156<br>Lysine_3.717_57.021<br>Tyrosine_7.181_133.38<br>Unknown_3.249_78.373<br>Unknown_3.406_78.52<br>Unknown_3.477_72.189<br>Unknown_3.712_72.274	Alanine_1.47_18.955<br>Alanine_3.772_53.166<br>Glycerol_3.772_74.329	Alanine_1.47_18.955<br>Alanine_3.772_53.166<br>Glucose_3.246_76.738<br>Glucose_3.538_74.093<br>Glucose_4.648_98.502<br>Glucose_5.234_94.693<br>Glycerol_3.772_74.329<br>Lactic_acid_4.109_71.139<br>Leucine_3.719_55.923<br>Lysine_1.72_29.156<br>Lysine_3.717_57.021<br>Tyrosine_7.181_133.38<br>Unknown_3.249_78.373<br>Unknown_3.477_72.189<br>Unknown_3.712_72.274	Age_at_Sample_Collection<br>Alanine_1.47_18.955<br>Alanine_3.772_53.166<br>Glucose_3.246_76.738<br>Glucose_3.538_74.093<br>Glucose_3.768_63.191<br>Glucose_3.831_63.217<br>Glucose_4.648_98.502<br>Glucose_5.234_94.693<br>Glycerol_3.772_74.329<br>Lactic_acid_4.109_71.139<br>Leucine_3.719_55.923<br>Lysine_1.72_29.156<br>Lysine_3.717_57.021 MoCA<br>Tyrosine_7.181_133.38 BMI	Alanine_1.47_18.955<br>Alanine_3.772_53.166<br>Glycerol_3.772_74.329<br>Lysine_3.717_57.021	Age_at_Sample_Collection<br>Alanine_1.47_18.955<br>Alanine_3.772_53.166<br>Glucose_3.246_76.738<br>Glucose_3.538_74.093<br>Glucose_3.768_63.191<br>Glucose_3.831_63.217<br>Glucose_4.648_98.502<br>Glucose_5.234_94.693<br>Glycerol_3.772_74.329<br>Lactic_acid_4.109_71.139<br>Leucine_3.719_55.923<br>Lysine_1.72_29.156<br>Lysine_3.717_57.021 MoCA<br>Tyrosine_7.181_133.38	Age_at_Sample_Collection<br>Alanine_1.47_18.955<br>Alanine_3.772_53.166<br>Glucose_3.246_76.738<br>Glucose_3.538_74.093<br>Glucose_3.768_63.191<br>Glucose_3.831_63.217<br>Glucose_4.648_98.502<br>Glucose_5.234_94.693<br>Glycerol_3.772_74.329<br>Lactic_acid_4.109_71.139<br>Leucine_3.719_55.923<br>Lysine_1.72_29.156<br>Lysine_3.717_57.021 MoCA<br>Tyrosine_7.181_133.38<br>Unknown_3.249_78.373<br>Unknown_3.406_78.52<br>Unknown_3.477_72.189<br>Unknown_3.712_72.274 BMI	Alanine_1.47_18.955<br>Alanine_3.772_53.166<br>Glycerol_3.772_74.329	Age_at_Sample_Collection<br>Alanine_1.47_18.955<br>Alanine_3.772_53.166<br>Glucose_3.246_76.738<br>Glucose_3.538_74.093<br>Glucose_3.768_63.191<br>Glucose_3.831_63.217<br>Glucose_4.648_98.502<br>Glucose_5.234_94.693<br>Glycerol_3.772_74.329<br>Lactic_acid_4.109_71.139<br>Leucine_3.719_55.923<br>Lysine_1.72_29.156<br>Lysine_3.717_57.021 MoCA<br>Tyrosine_7.181_133.38<br>Unknown_3.249_78.373<br>Unknown_3.406_78.52<br>Unknown_3.477_72.189<br>Unknown_3.712_72.274

Table S7. Cross-validation ML Model Performance

feature_sel	feature_filter	model	metrics	group1	group2	p	mean_train	mean_test	sd_train	sd_test
KW-only	Known	OPLS-DA	Accuracy	train	test	0.097432	0.775472	0.733333	0.024277	0.055895
KW-only	Known	OPLS-DA	AUC	train	test	0.1473	0.771739	0.727778	0.02591	0.070273
KW-only	Known	OPLS-DA	Precision	train	test	0.076677	0.740285	0.694295	0.026421	0.060222
KW-only	Known	OPLS-DA	Sensitivity	train	test	0.458178	0.743478	0.688889	0.043236	0.194577
KW-only	Known	OPLS-DA	Specificity	train	test	0.282894	0.8	0.766667	0.022222	0.086066
KW-only	Known	RF	Accuracy	train	test	0.461823	0.615094	0.647619	0.051249	0.095766
KW-only	Known	RF	AUC	train	test	0.059069	0.643696	0.752315	0.077287	0.087902
KW-only	Known	RF	Precision	train	test	0.599622	0.565886	0.591533	0.069688	0.100833
KW-only	Known	RF	Sensitivity	train	test	0.980873	0.513043	0.511111	0.057242	0.210819
KW-only	Known	RF	Specificity	train	test	0.197914	0.693333	0.75	0.069921	0.078567
KW-Boruta	Known	OPLS-DA	Accuracy	train	test	0.506168	0.743396	0.714286	0.051249	0.089791
KW-Boruta	Known	OPLS-DA	AUC	train	test	0.463799	0.733768	0.7	0.055663	0.094924
KW-Boruta	Known	OPLS-DA	Precision	train	test	0.634521	0.721802	0.69316	0.059404	0.133375
KW-Boruta	Known	OPLS-DA	Sensitivity	train	test	0.392802	0.66087	0.6	0.095696	0.158871
KW-Boruta	Known	OPLS-DA	Specificity	train	test	0.871562	0.806667	0.8	0.037843	0.097816
KW-Boruta	Known	RF	Accuracy	train	test	0.429968	0.666038	0.619048	0.065993	0.116642
KW-Boruta	Known	RF	AUC	train	test	0.656605	0.721087	0.690278	0.072753	0.150199
KW-Boruta	Known	RF	Precision	train	test	0.531817	0.622919	0.573214	0.078568	0.167529
KW-Boruta	Known	RF	Sensitivity	train	test	0.993902	0.578261	0.577778	0.094036	0.136586
KW-Boruta	Known	RF	Specificity	train	test	0.248219	0.733333	0.65	0.054433	0.17033
KW-RFE	Known	OPLS-DA	Accuracy	train	test	0.016981	0.790566	0.685714	0.030097	0.087518
KW-RFE	Known	OPLS-DA	AUC	train	test	0.013346	0.78913	0.679167	0.031401	0.085979
KW-RFE	Known	OPLS-DA	Precision	train	test	0.058198	0.749758	0.646389	0.036046	0.122461
KW-RFE	Known	OPLS-DA	Sensitivity	train	test	0.016651	0.778261	0.633333	0.052053	0.117706
KW-RFE	Known	OPLS-DA	Specificity	train	test	0.141708	0.8	0.725	0.035136	0.124536
KW-RFE	Known	RF	Accuracy	train	test	0.821249	0.641509	0.652381	0.06536	0.087085
KW-RFE	Known	RF	AUC	train	test	0.99435	0.697754	0.698148	0.061195	0.117928
KW-RFE	Known	RF	Precision	train	test	0.768922	0.600949	0.62149	0.08783	0.137906
KW-RFE	Known	RF	Sensitivity	train	test	0.951897	0.526087	0.522222	0.092686	0.128834
KW-RFE	Known	RF	Specificity	train	test	0.71155	0.73	0.75	0.074453	0.117851
KW-RFE	Known_Unknown_Clinical	RF	Specificity	train	test	0.706637	0.73	0.741667	0.067495	0.082869

Table S7. Cross-validation ML Model Performance (continued)

KW-only	Known_Clinical	OPLS-DA	Accuracy	train	test	0.004644	0.790566	0.695238	0.030097	0.055895
KW-only	Known_Clinical	OPLS-DA	AUC	train	test	0.001986	0.78913	0.681944	0.029676	0.055343
KW-only	Known_Clinical	OPLS-DA	Precision	train	test	0.059132	0.750769	0.669048	0.042099	0.088609
KW-only	Known_Clinical	OPLS-DA	Sensitivity	train	test	2.90E-04	0.778261	0.588889	0.043236	0.091475
KW-only	Known_Clinical	OPLS-DA	Specificity	train	test	0.512941	0.8	0.775	0.044444	0.088279
KW-only	Known_Clinical	RF	Accuracy	train	test	0.026476	0.667925	0.62381	0.02547	0.047354
KW-only	Known_Clinical	RF	AUC	train	test	0.065072	0.729203	0.676389	0.036632	0.056383
KW-only	Known_Clinical	RF	Precision	train	test	0.114	0.630287	0.581429	0.04325	0.068584
KW-only	Known_Clinical	RF	Sensitivity	train	test	0.032159	0.578261	0.444444	0.041247	0.148148
KW-only	Known_Clinical	RF	Specificity	train	test	0.593267	0.736667	0.758333	0.053171	0.082869
KW-Boruta	Known_Clinical	OPLS-DA	Accuracy	train	test	0.925816	0.726415	0.72381	0.04557	0.062693
KW-Boruta	Known_Clinical	OPLS-DA	AUC	train	test	0.866985	0.712174	0.716667	0.049489	0.062182
KW-Boruta	Known_Clinical	OPLS-DA	Precision	train	test	0.574388	0.719689	0.695184	0.054975	0.099036
KW-Boruta	Known_Clinical	OPLS-DA	Sensitivity	train	test	0.089565	0.604348	0.666667	0.088037	0.117121
KW-Boruta	Known_Clinical	OPLS-DA	Specificity	train	test	0.22523	0.82	0.766667	0.039126	0.109713
KW-Boruta	Known_Clinical	RF	Accuracy	train	test	0.113374	0.666038	0.714286	0.04626	0.063492
KW-Boruta	Known_Clinical	RF	AUC	train	test	0.035652	0.700217	0.789352	0.051673	0.068954
KW-Boruta	Known_Clinical	RF	Precision	train	test	0.280278	0.623069	0.6575	0.049899	0.065957
KW-Boruta	Known_Clinical	RF	Sensitivity	train	test	0.041799	0.578261	0.7	0.096243	0.139074
KW-Boruta	Known_Clinical	RF	Specificity	train	test	0.798837	0.733333	0.725	0.044444	0.079057
KW-RFE	Known_Clinical	OPLS-DA	Accuracy	train	test	0.277979	0.771698	0.72381	0.045744	0.089228
KW-RFE	Known_Clinical	OPLS-DA	AUC	train	test	0.406442	0.76587	0.726389	0.047799	0.098438
KW-RFE	Known_Clinical	OPLS-DA	Precision	train	test	0.047111	0.743782	0.655935	0.049788	0.082026
KW-RFE	Known_Clinical	OPLS-DA	Sensitivity	train	test	0.777327	0.721739	0.744444	0.065458	0.189215
KW-RFE	Known_Clinical	OPLS-DA	Specificity	train	test	0.008109	0.81	0.708333	0.035312	0.09001
KW-RFE	Known_Clinical	RF	Accuracy	train	test	0.423593	0.645283	0.685714	0.056813	0.108123
KW-RFE	Known_Clinical	RF	AUC	train	test	0.399012	0.689493	0.742593	0.067643	0.126208
KW-RFE	Known_Clinical	RF	Precision	train	test	0.743306	0.605861	0.625159	0.071485	0.131967
KW-RFE	Known_Clinical	RF	Sensitivity	train	test	0.134643	0.517391	0.655556	0.097112	0.199106
KW-RFE	Known_Clinical	RF	Specificity	train	test	0.271246	0.743333	0.708333	0.054546	0.105775

Table S7. Cross-validation ML Model Performance (continued)

KW-only	Known_Unknown	OPLS-DA	Accuracy	train	test	0.679258	0.769811	0.752381	0.046045	0.086359
KW-only	Known_Unknown	OPLS-DA	AUC	train	test	0.666924	0.768261	0.75	0.049263	0.085867
KW-only	Known_Unknown	OPLS-DA	Precision	train	test	0.848688	0.724131	0.713763	0.042723	0.124889
KW-only	Known_Unknown	OPLS-DA	Sensitivity	train	test	0.666536	0.756522	0.733333	0.077234	0.119441
KW-only	Known_Unknown	OPLS-DA	Specificity	train	test	0.77912	0.78	0.766667	0.032203	0.116534
KW-only	Known_Unknown	RF	Accuracy	train	test	0.190636	0.607547	0.661905	0.033041	0.101513
KW-only	Known_Unknown	RF	AUC	train	test	0.097706	0.669493	0.713889	0.053307	0.03046
KW-only	Known_Unknown	RF	Precision	train	test	0.278494	0.557398	0.625278	0.048284	0.158847
KW-only	Known_Unknown	RF	Sensitivity	train	test	0.060045	0.491304	0.577778	0.05442	0.102104
KW-only	Known_Unknown	RF	Specificity	train	test	0.567895	0.696667	0.725	0.063732	0.124536
KW-Boruta	Known_Unknown	OPLS-DA	Accuracy	train	test	0.506168	0.743396	0.714286	0.051249	0.089791
KW-Boruta	Known_Unknown	OPLS-DA	AUC	train	test	0.463799	0.733768	0.7	0.055663	0.094924
KW-Boruta	Known_Unknown	OPLS-DA	Precision	train	test	0.634521	0.721802	0.69316	0.059404	0.133375
KW-Boruta	Known_Unknown	OPLS-DA	Sensitivity	train	test	0.392802	0.66087	0.6	0.095696	0.158871
KW-Boruta	Known_Unknown	OPLS-DA	Specificity	train	test	0.871562	0.806667	0.8	0.037843	0.097816
KW-Boruta	Known_Unknown	RF	Accuracy	train	test	0.429968	0.666038	0.619048	0.065993	0.116642
KW-Boruta	Known_Unknown	RF	AUC	train	test	0.656605	0.721087	0.690278	0.072753	0.150199
KW-Boruta	Known_Unknown	RF	Precision	train	test	0.531817	0.622919	0.573214	0.078568	0.167529
KW-Boruta	Known_Unknown	RF	Sensitivity	train	test	0.993902	0.578261	0.577778	0.094036	0.136586
KW-Boruta	Known_Unknown	RF	Specificity	train	test	0.248219	0.733333	0.65	0.054433	0.17033
KW-RFE	Known_Unknown	OPLS-DA	Accuracy	train	test	0.21853	0.773585	0.738095	0.030811	0.060443
KW-RFE	Known_Unknown	OPLS-DA	AUC	train	test	0.181119	0.772101	0.733333	0.032152	0.061698
KW-RFE	Known_Unknown	OPLS-DA	Precision	train	test	0.409467	0.729236	0.697803	0.03308	0.084648
KW-RFE	Known_Unknown	OPLS-DA	Sensitivity	train	test	0.122827	0.76087	0.7	0.046962	0.105409
KW-RFE	Known_Unknown	OPLS-DA	Specificity	train	test	0.640719	0.783333	0.766667	0.028328	0.086066
KW-RFE	Known_Unknown	RF	Accuracy	train	test	0.990036	0.643396	0.642857	0.064416	0.09049
KW-RFE	Known_Unknown	RF	AUC	train	test	0.456733	0.68	0.713889	0.040531	0.11072
KW-RFE	Known_Unknown	RF	Precision	train	test	0.890523	0.601596	0.60943	0.081122	0.127959
KW-RFE	Known_Unknown	RF	Sensitivity	train	test	0.62886	0.534783	0.555556	0.082111	0.117121
KW-RFE	Known_Unknown	RF	Specificity	train	test	0.727555	0.726667	0.708333	0.068132	0.148293

Table S7. Cross-validation ML Model Performance (continued)

KW-only	Known_Unknown_Clinical	OPLS-DA	Accuracy	train	test	0.035021	0.8	0.685714	0.046388	0.105767
KW-only	Known_Unknown_Clinical	OPLS-DA	AUC	train	test	0.027733	0.798478	0.679167	0.048992	0.1032
KW-only	Known_Unknown_Clinical	OPLS-DA	Precision	train	test	0.077496	0.75996	0.654423	0.046166	0.125013
KW-only	Known_Unknown_Clinical	OPLS-DA	Sensitivity	train	test	0.032929	0.786957	0.633333	0.072319	0.157571
KW-only	Known_Unknown_Clinical	OPLS-DA	Specificity	train	test	0.2068	0.81	0.725	0.035312	0.166898
KW-only	Known_Unknown_Clinical	RF	Accuracy	train	test	0.199975	0.65283	0.604762	0.031067	0.084142
KW-only	Known_Unknown_Clinical	RF	AUC	train	test	0.700849	0.697609	0.674537	0.049876	0.143012
KW-only	Known_Unknown_Clinical	RF	Precision	train	test	0.424838	0.618111	0.575094	0.03907	0.133018
KW-only	Known_Unknown_Clinical	RF	Sensitivity	train	test	0.130958	0.521739	0.466667	0.061488	0.102104
KW-only	Known_Unknown_Clinical	RF	Specificity	train	test	0.428871	0.753333	0.708333	0.032203	0.163158
KW-Boruta	Known_Unknown_Clinical	OPLS-DA	Accuracy	train	test	0.506168	0.743396	0.714286	0.051249	0.089791
KW-Boruta	Known_Unknown_Clinical	OPLS-DA	AUC	train	test	0.463799	0.733768	0.7	0.055663	0.094924
KW-Boruta	Known_Unknown_Clinical	OPLS-DA	Precision	train	test	0.634521	0.721802	0.69316	0.059404	0.133375
KW-Boruta	Known_Unknown_Clinical	OPLS-DA	Sensitivity	train	test	0.392802	0.66087	0.6	0.095696	0.158871
KW-Boruta	Known_Unknown_Clinical	OPLS-DA	Specificity	train	test	0.871562	0.806667	0.8	0.037843	0.097816
KW-Boruta	Known_Unknown_Clinical	RF	Accuracy	train	test	0.429968	0.666038	0.619048	0.065993	0.116642
KW-Boruta	Known_Unknown_Clinical	RF	AUC	train	test	0.656605	0.721087	0.690278	0.072753	0.150199
KW-Boruta	Known_Unknown_Clinical	RF	Precision	train	test	0.531817	0.622919	0.573214	0.078568	0.167529
KW-Boruta	Known_Unknown_Clinical	RF	Sensitivity	train	test	0.993902	0.578261	0.577778	0.094036	0.136586
KW-Boruta	Known_Unknown_Clinical	RF	Specificity	train	test	0.248219	0.733333	0.65	0.054433	0.17033
KW-RFE	Known_Unknown_Clinical	OPLS-DA	Accuracy	train	test	0.017655	0.79434	0.695238	0.039226	0.075125
KW-RFE	Known_Unknown_Clinical	OPLS-DA	AUC	train	test	0.014815	0.790435	0.680556	0.043137	0.08125
KW-RFE	Known_Unknown_Clinical	OPLS-DA	Precision	train	test	0.065839	0.764179	0.672817	0.036555	0.106211
KW-RFE	Known_Unknown_Clinical	OPLS-DA	Sensitivity	train	test	0.017962	0.76087	0.577778	0.080039	0.155379
KW-RFE	Known_Unknown_Clinical	OPLS-DA	Specificity	train	test	0.324825	0.82	0.783333	0.032203	0.089581
KW-RFE	Known_Unknown_Clinical	RF	Accuracy	train	test	0.705171	0.635849	0.657143	0.067181	0.11621
KW-RFE	Known_Unknown_Clinical	RF	AUC	train	test	0.310432	0.671812	0.721759	0.05814	0.095341
KW-RFE	Known_Unknown_Clinical	RF	Precision	train	test	0.942727	0.593888	0.589138	0.085359	0.132794
KW-RFE	Known_Unknown_Clinical	RF	Sensitivity	train	test	0.760665	0.513043	0.544444	0.084008	0.248176

Table S8. Sensitivity Analysis of Champion Model

Model Based on Champion Model	AUC*	Accuracy*	Sensitivity*	Specificity*	Precision*
no_alanine	60.8% (-14.2%)	61.4% (-13.8%)	56.7% (-16.6%)	65.0% (-11.7%)	54.7% (-16.7%)
no_glucose	65.4% (-9.6%)	65.7% (-9.5%)	63.3% (-10.0%)	67.5% (-9.2%)	59.4% (-12.0%)
no_glycerol	71.4% (-3.6%)	72.4% (-2.8%)	64.4% (-8.9%)	78.3% (1.6%)	71.3% (-0.1%)
no_lactic_acid	70.0% (-5.0%)	70.5% (-4.7%)	66.7% (-6.6%)	73.3% (-3.4%)	66.5% (-4.9%)
no_leucine	72.1% (-2.9%)	72.4% (-2.8%)	70.0% (-3.3%)	74.2% (-2.5%)	69.8% (-1.6%)
no_lysine	64.6% (-10.4%)	64.8% (-10.4%)	58.9% (-14.4%)	69.2% (-7.5%)	62.8% (-8.6%)
no_tyrosine	69.9% (-5.1%)	71.0% (-4.2%)	62.2% (-11.1%)	77.5% (0.8%)	67.3% (-4.1%)

*: model mean performance metric value with difference compared to champion model in parenthesis

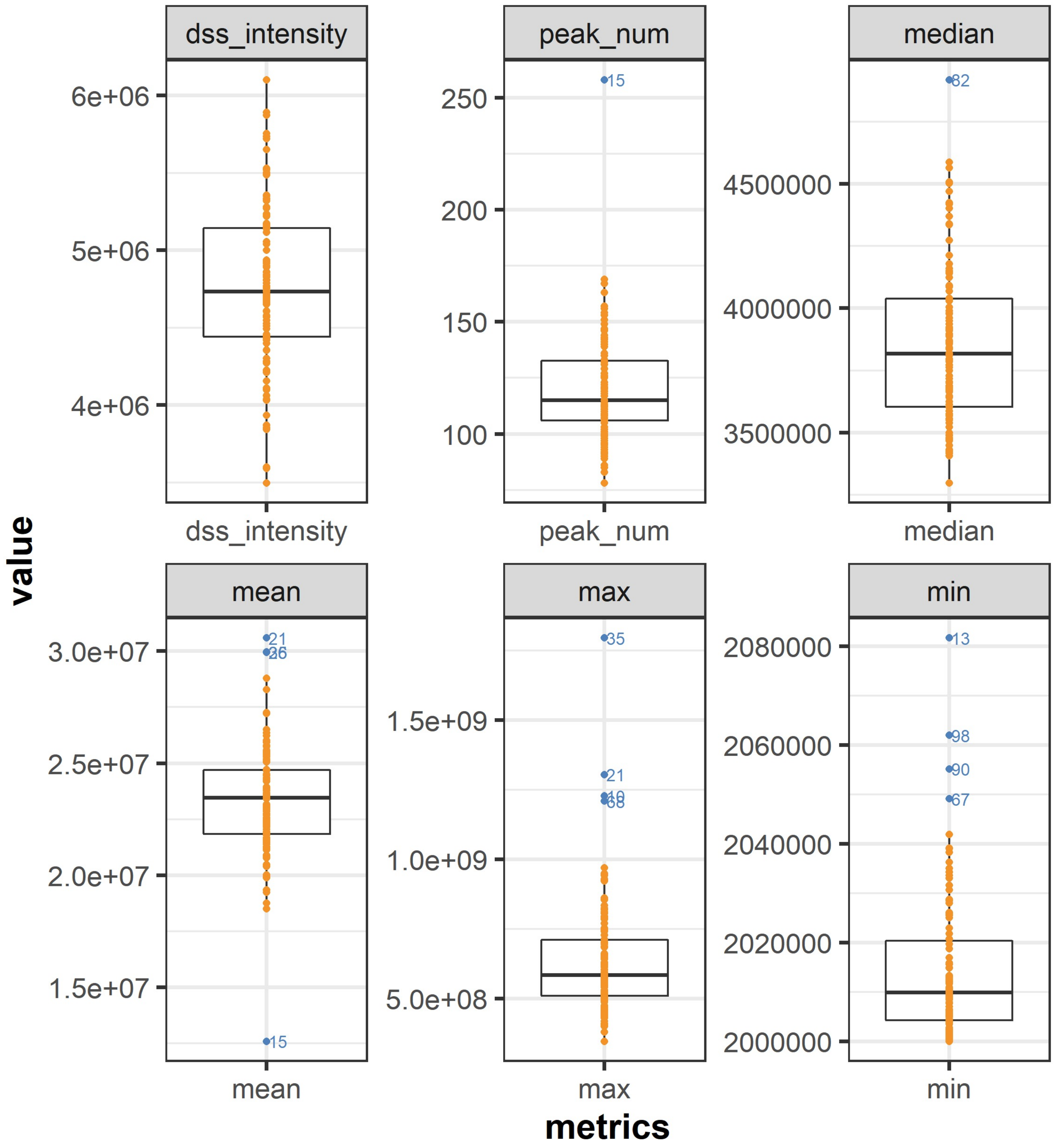

Figure S1: NMR data quality control and assurance by measuring the mean, median, maximum and minimum intensity values and number of metabolite resonances detected. Patient id 15 was excluded as having both a significantly higher number of peaks and significantly lower mean intensity value.

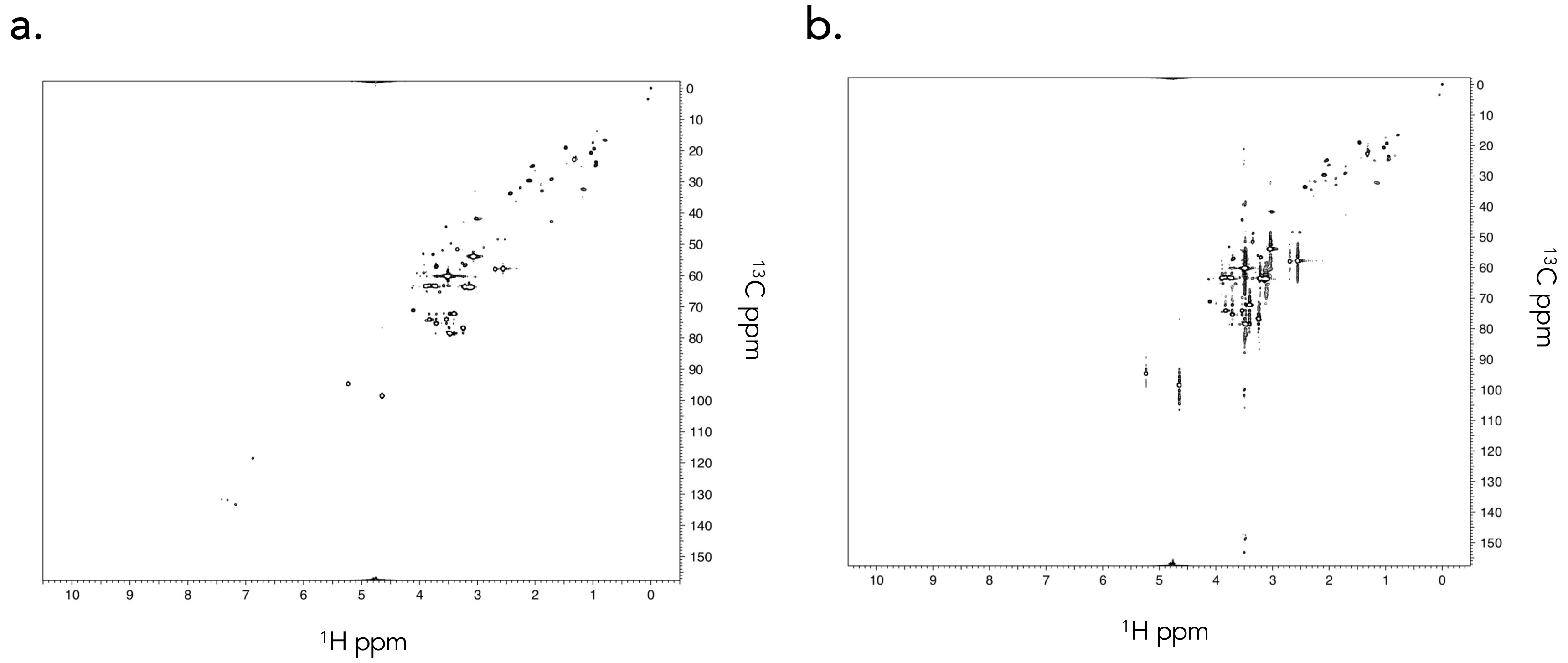

Figure S2: NMR spectral comparison, a) NMR spectrum from a representative sample compared with **b)** outlier (patient ID 15) sample spectrum. Both spectrum were drawn at simmilar s/n ratio for comparison. NMR spectrum for outlier showed severe streaking in F1 dimension especially between 50-100 ppm region.

a.

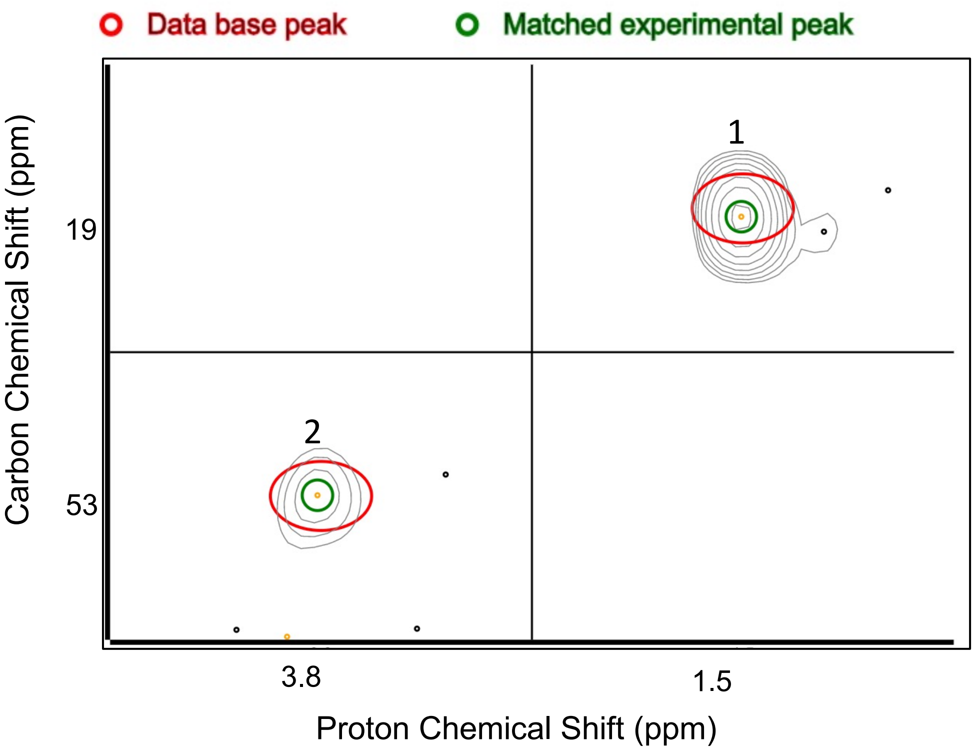

Alanine

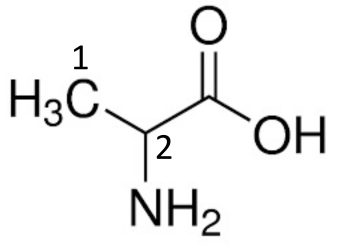

Position	Database chemical shift (ppm) ^1H	Database chemical shift (ppm) ^{13}C	Experimental chemical shift (ppm) ^1H	Experimental chemical shift (ppm) ^{13}C
1	1.47	18.87	1.47	18.96
2	3.77	53.22	3.77	53.16

b.

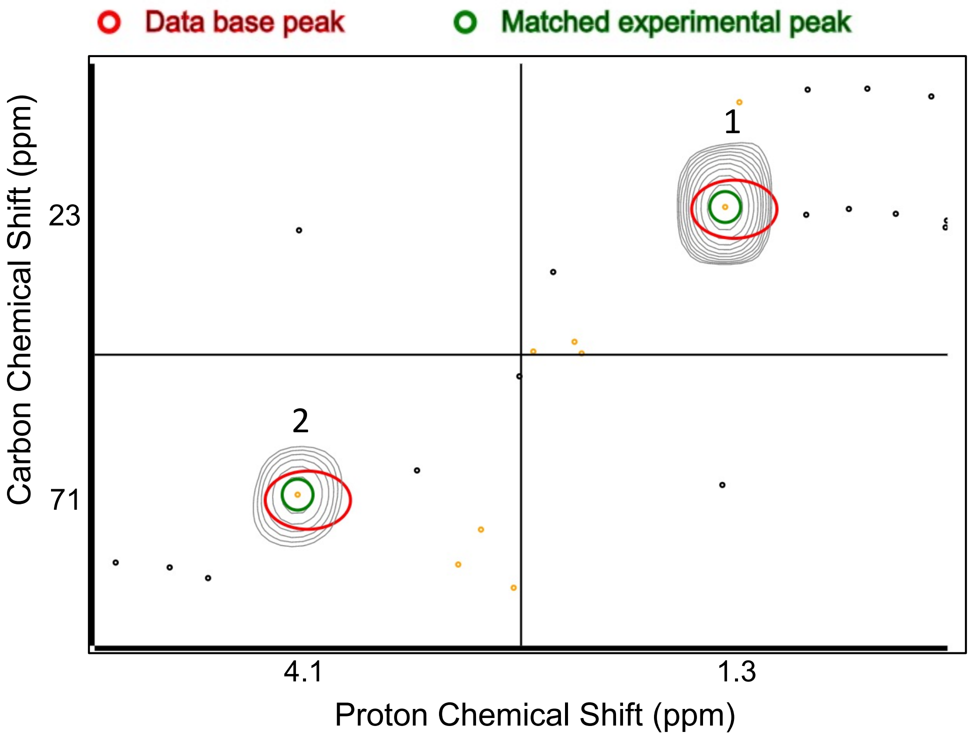

Lactic acid

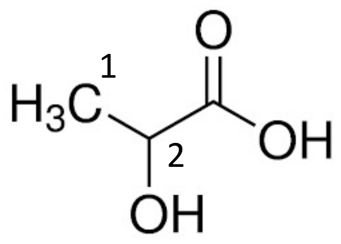

Position	Database chemical shift (ppm) ^1H	Database chemical shift (ppm) ^{13}C	Experimental chemical shift (ppm) ^1H	Experimental chemical shift (ppm) ^{13}C
1	1.32	22.81	1.32	22.75
2	4.10	71.23	4.11	71.13

c.

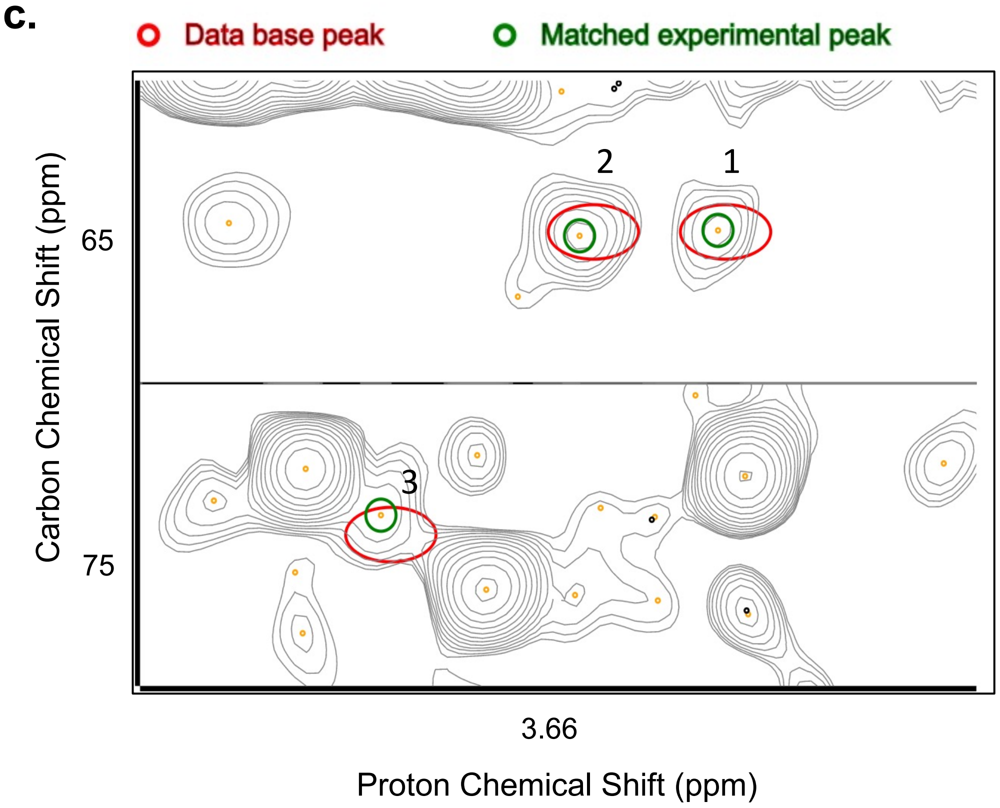

Glycerol

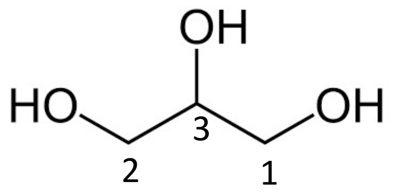

Position	Database chemical shift (ppm) ^1H	Database chemical shift (ppm) ^{13}C	Experimental chemical shift (ppm) ^1H	Experimental chemical shift (ppm) ^{13}C
1	3.55	65.22	3.55	65.20
2	3.64	65.22	3.65	65.26
3	3.77	74.81	3.78	74.60

Figure S3: NMR annotations for top differential features. Example NMR spectral resonances (left) and corresponding chemical shifts (right) for, **a)** alanine, **b)** lactic acid**, c)** glycerol **d)** Lysine **d)** Leucine **d)** Tyrosine **e)** Glucose and **f)** Glutamine

Cont....

d.

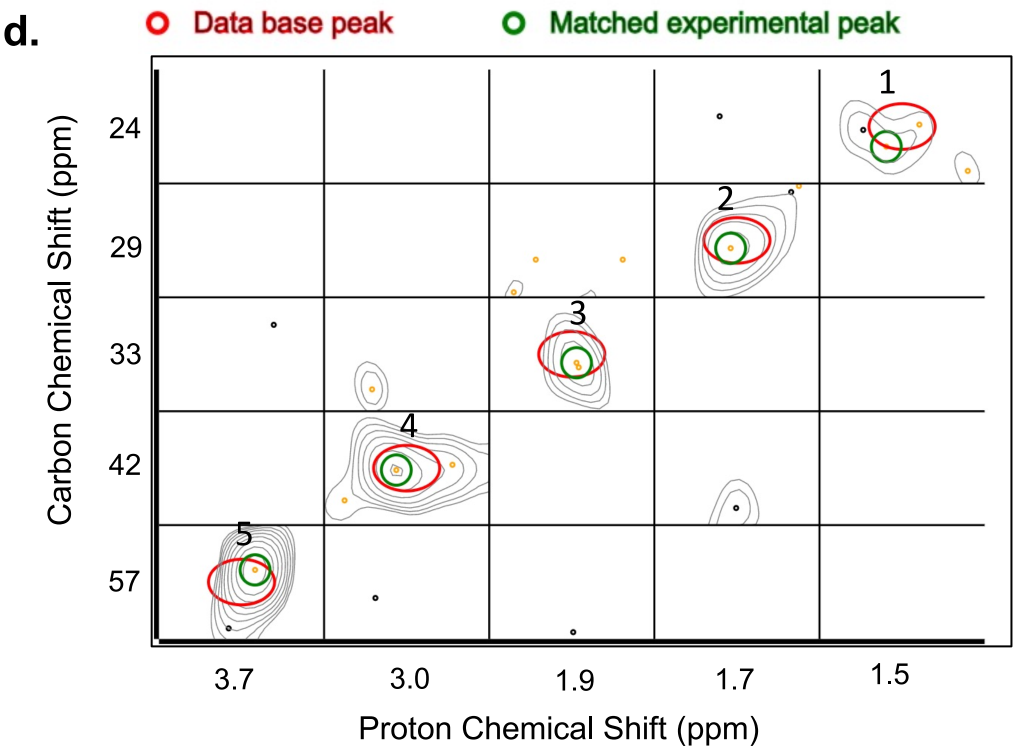

Lysine

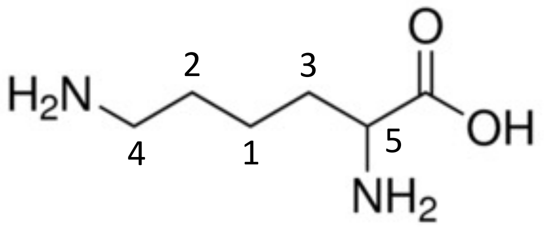

Position	Database chemical shift (ppm)		Experimental chemical shift (ppm)	
	^{1}H	^{13}C	^{1}H	^{13}C
1	1.46	24.14	1.48	24.41
2	1.72	29.14	1.72	29.24
3	1.90	32.63	1.89	32.74
4	3.01	41.76	3.02	41.78
5	3.75	57.19	3.73	57.03

e.

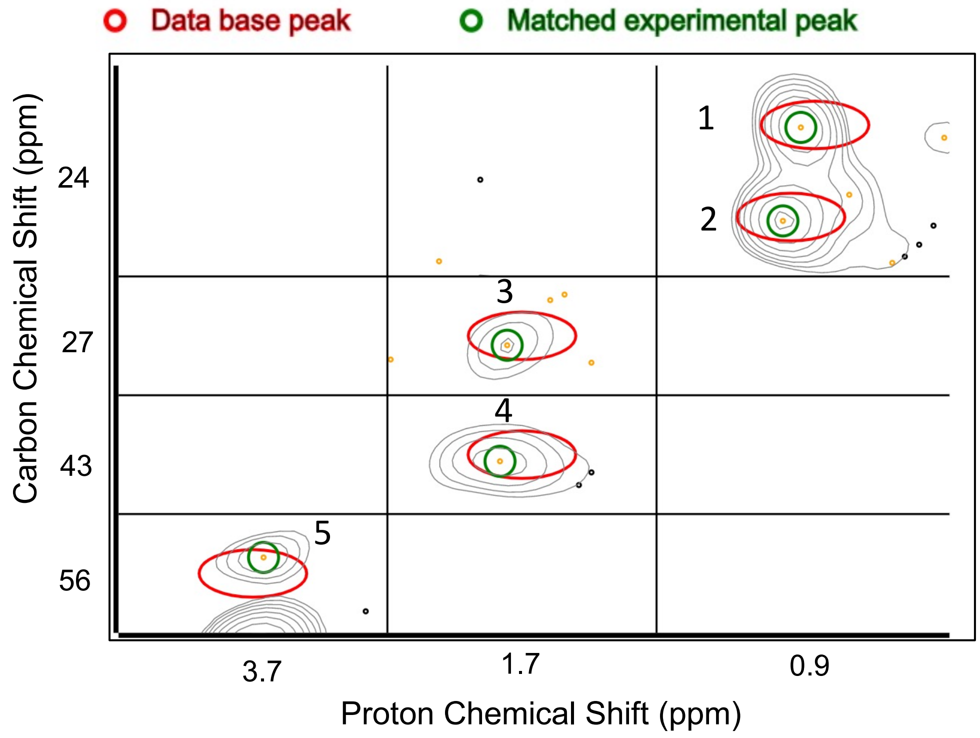

Leucine

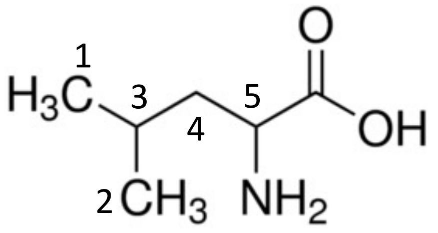

Position	Database chemical shift (ppm)		Experimental chemical shift (ppm)	
	^{1}H	^{13}C	^{1}H	^{13}C
1	0.94	23.59	0.95	23.62
2	0.96	24.75	0.96	24.80
3	1.70	26.87	1.71	26.99
4	1.70	42.53	1.72	42.61
5	3.72	56.11	3.72	55.91

f.

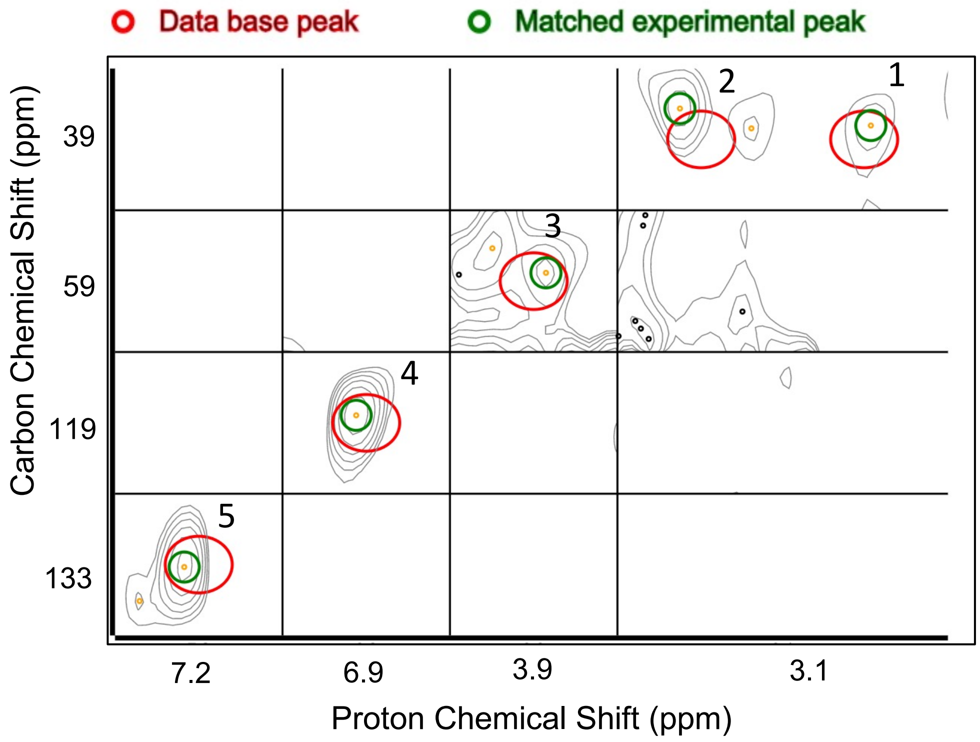

Tyrosine

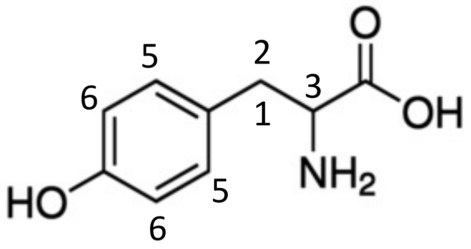

Position	Database chemical shift (ppm)		Experimental chemical shift (ppm)	
	^{1}H	^{13}C	^{1}H	^{13}C
1	3.02	38.39	3.02	38.24
2	3.17	38.39	3.19	38.06
3	3.92	59.00	3.91	58.91
4	6.88	118.63	6.89	118.55
5	7.17	133.45	7.18	133.48

Figure S3: NMR annotations for top differential features. Example NMR spectral resonances (left) and corresponding chemical shifts (right) for, **a)** alanine, **b)** lactic acid**, c)** glycerol **d)** Lysine **d)** Leucine **d)** Tyrosine **e)** Glucose and **f)** Glutamine

g.

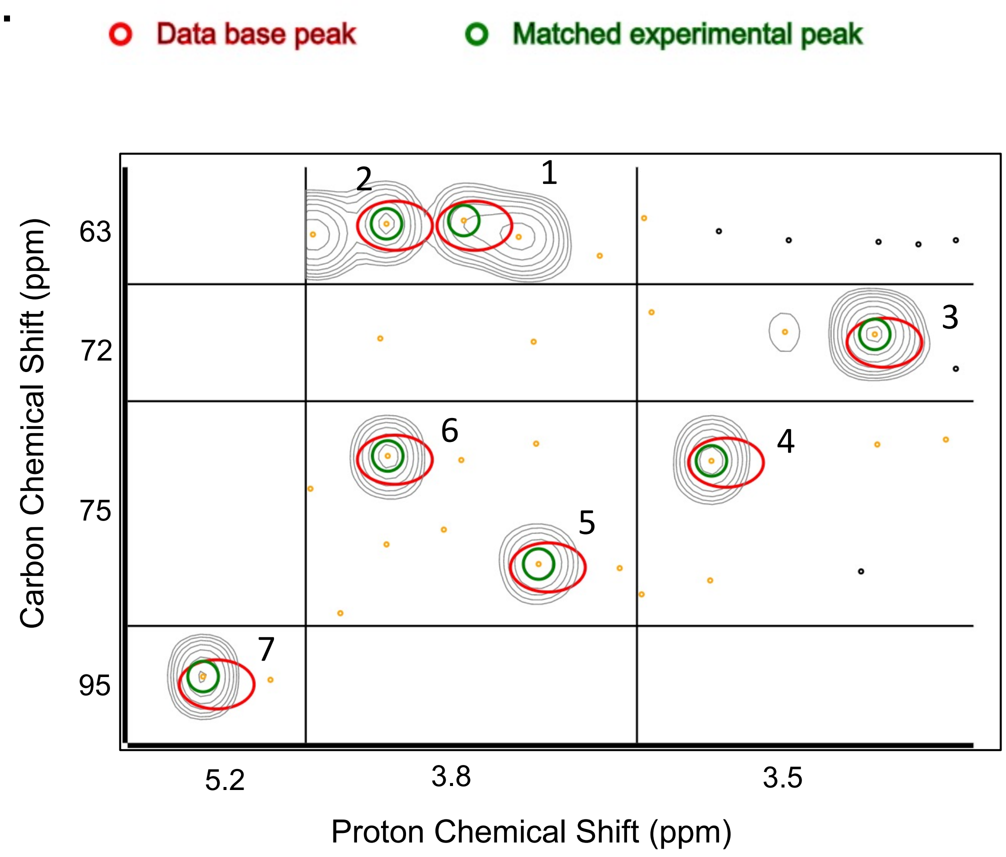

Glucose

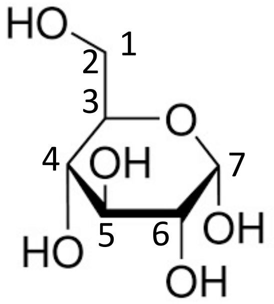

Position	Database chemical shift (ppm)		Experimental chemical shift (ppm)	
	^{1}H	^{13}C	^{1}H	^{13}C
1	3.76	63.28	3.77	63.22
2	3.82	63.28	3.83	63.26
3	3.40	72.35	3.40	72.25
4	3.52	74.20	3.54	74.18
5	3.70	75.48	3.71	75.44
6	3.82	74.17	3.83	74.12
7	5.22	94.83	5.23	94.73

e.

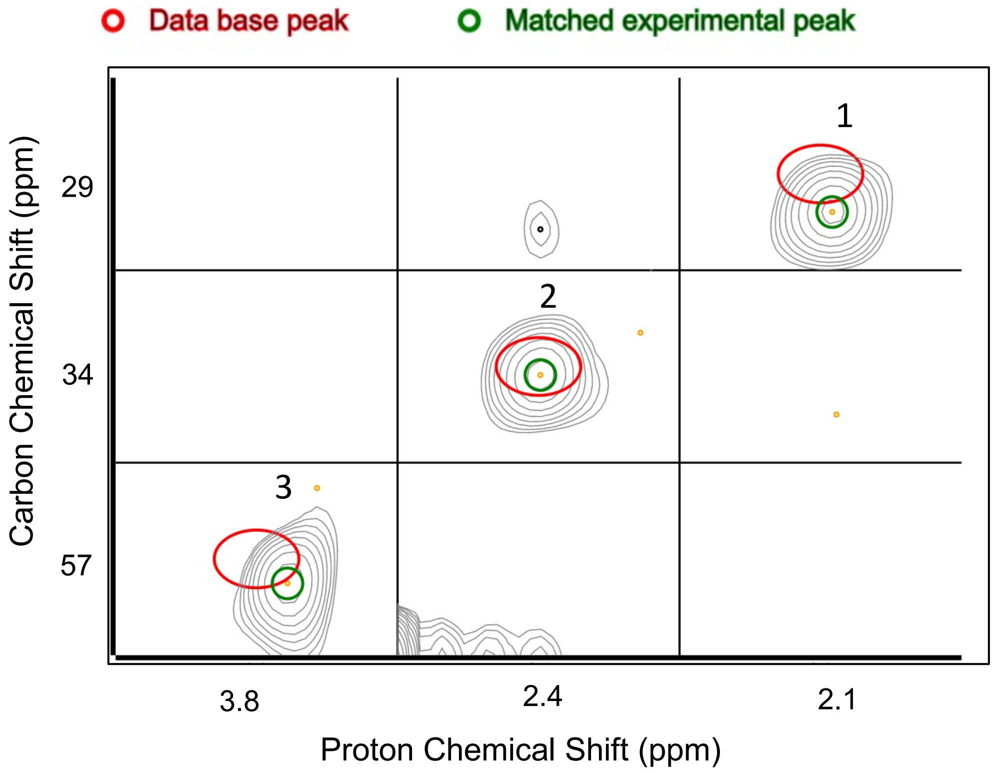

Glutamine

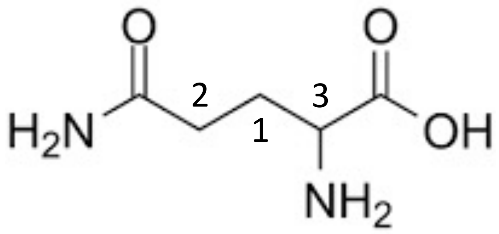

Position	Database chemical shift (ppm)		Experimental chemical shift (ppm)	
	^{1}H	^{13}C	^{1}H	^{13}C
1	2.13	28.95	2.12	29.34
2	2.44	33.52	2.44	33.61
3	3.76	56.83	3.74	57.08

Figure S3: NMR annotations for top differential features. Example NMR spectral resonances (left) and corresponding chemical shifts (right) for, **a)** alanine, **b)** lactic acid**, c)** glycerol **d)** Lysine **d)** Leucine **d)** Tyrosine **e)** Glucose and **f)** Glutamine

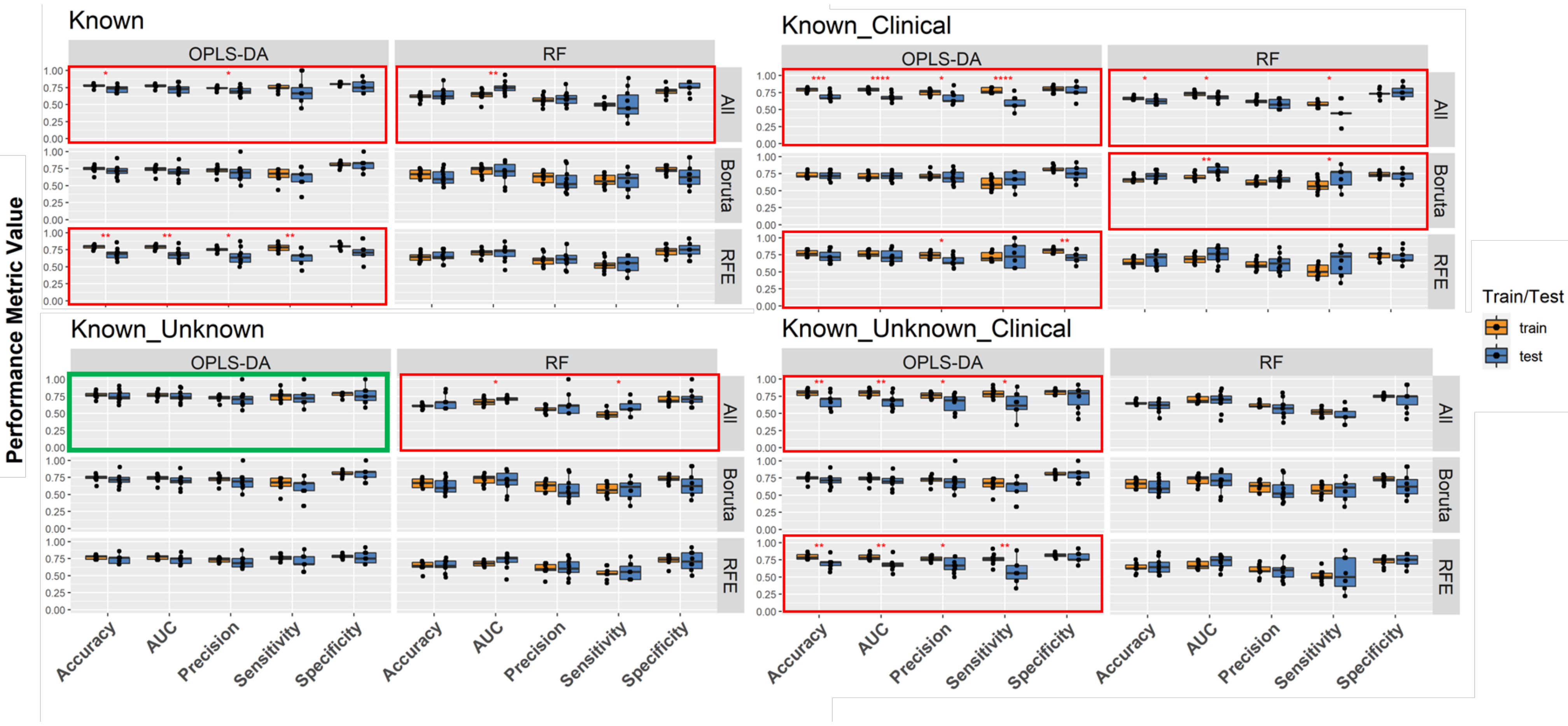

Figure S4: Performance of champion machine learning algorithm for LRRK2 PD classification. The data was split intro 70/30 training and test data, 10x cross-validated using with 4 predefined feature sets and 3 feature selection methods, and 3 machine learning models. Models with red square were excluded due to potential overfitting. The champion model with green square is selected based on the testing AUC.

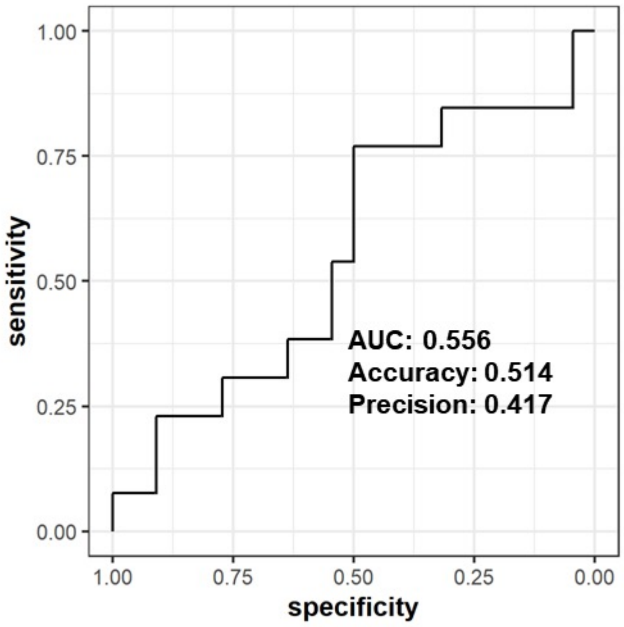

Figure S5: Receiver operator characteristic (ROC) analysis with 0.556 area under the curve (AUC) using male BoR score to predict on female cohort.
